# IgG Fc-Binding Peptide-Conjugated Pan-CoV Fusion Inhibitor Exhibits Extended In Vivo Half-Life and Synergistic Antiviral Effect When Combined with Neutralizing Antibodies

**DOI:** 10.3390/biom13091283

**Published:** 2023-08-22

**Authors:** Xiaojie Su, Ziqi Huang, Wei Xu, Qian Wang, Lixiao Xing, Lu Lu, Shibo Jiang, Shuai Xia

**Affiliations:** Key Laboratory of Medical Molecular Virology (MOE/NHC/CAMS), School of Basic Medical Sciences, Shanghai Institute of Infectious Disease and Biosecurity, Shanghai Frontiers Science Center of Pathogenic Microbes and Infection, Fudan University, Shanghai 200032, China; 17111010016@fudan.edu.cn (X.S.); 22111010065@fudan.edu.cn (Z.H.); xuwei11@fudan.edu.cn (W.X.); wang_qian@fudan.edu.cn (Q.W.); 20111010065@fudan.edu.cn (L.X.); lul@fudan.edu.cn (L.L.)

**Keywords:** human coronavirus, fusion inhibitor, IgG-binding peptide, long-acting strategy, half-life, neutralizing antibodies

## Abstract

The peptide-based pan-coronavirus fusion inhibitor EK1 is in phase III clinical trials, and it has, thus far, shown good clinical application prospects against SARS-CoV-2 and its variants. To further improve its in vivo long-acting property, we herein developed an Fc-binding strategy by conjugating EK1 with human immunoglobulin G Fc-binding peptide (IBP), which can exploit the long half-life advantage of IgG in vivo. The newly engineered peptide IBP-EK1 showed potent and broad-spectrum inhibitory activity against SARS-CoV-2 and its variants, including various Omicron sublineages and other human coronaviruses (HCoVs) with low cytotoxicity. In mouse models, IBP-EK1 possessed potent prophylactic and therapeutic efficacy against lethal HCoV-OC43 challenge, and it showed good safety profile and low immunogenicity. More importantly, IBP-EK1 exhibited a significantly extended in vivo half-life in rhesus monkeys of up to 37.7 h, which is about 20-fold longer than that reported for EK1. Strikingly, IBP-EK1 displayed strong in vitro or ex vivo synergistic anti-HCoV effect when combined with monoclonal neutralizing antibodies, including REGN10933 or S309, suggesting that IBP-conjugated EK1 can be further developed as a long-acting, broad-spectrum anti-HCoV agent, either alone or in combination with neutralizing antibodies, to combat the current COVID-19 pandemic or future outbreaks caused by emerging and re-emerging highly pathogenic HCoVs.

## 1. Introduction

Coronaviruses (CoVs) are widely distributed, zoonotic enveloped viruses with a positive-sense, single-stranded RNA genome. CoVs can be divided into four major genera: alphacoronavirus (α-CoV), betacoronavirus (β-CoV), gammacoronavirus (γ-CoV), and deltacoronavirus (δ-CoV) [1]. Nine known coronaviruses can infect humans (HCoVs) [2]. Among them, HCoV-NL63 and HCoV-229E of α-CoV and HCoV-HKU1 and HCoV-OC43 of β-CoV can cause mild upper respiratory symptoms in healthy adults. Nonetheless, these HCoVs are responsible for serious infections, even life-threatening infections in infants and elderly or immunocompromised individuals [3,4]. Three highly pathogenic HCoVs belong to β-CoV, including SARS-CoV, MERS-CoV, and SARS-CoV-2 [5,6,7], resulting in severe respiratory syndrome in humans. Another two HCoVs, Canine Coronavirus-human pneumonia-2018 (CCoV-HuPn-2018) of α-CoV [8,9] and Hu-PDCoV of δ-CoV [10], were also newly identified in recent years. In addition, different SARS-like coronaviruses (SL-CoVs) have been isolated from bats [11], such as SHC014-CoV and WIV1-CoV [12,13], belonging to the Sarbecovirus subgenus of β-CoV, along with SARS-CoV and SARS-CoV-2 [14]. Studies showed that divergent CoVs in bats are infectious to human cells and pose a spillover risk to humans [15,16], suggesting the probability of emergent HCoVs in the future. Since the outbreak of SARS-CoV-2 in 2019 [17], dominant variants have evolved continuously. After successive infection waves caused by Alpha, Beta, Gamma, and Delta variants of concern (VOCs) [18], Omicron then quickly dominated from late 2021 and evolved into different sublineages, including BA.1-BA.5, BF.7, BQ.1.1, and XBB [18,19]. Despite relatively mild symptoms [20], Omicron infection is far more transmissible compared with previous VOCs [21]. In addition, with its dramatic immune escape from neutralizing antibodies [22], emergency use authorizations (EUAs) for several approved therapies of monoclonal neutralizing antibodies have been abolished [21], such as bamlanivimab/etesevimab (LY-CoV555/LY-CoV016), casirivimab/imdevimab (REGN10933/REGN10987), and sotrovimab, an optimized form of S309. This calls for haste in the development of broad-spectrum anti-HCoV agents in order to combat the current Omicron pandemic and the real possibility of other emerging HCoV outbreaks in the future.

**Figure 1:**
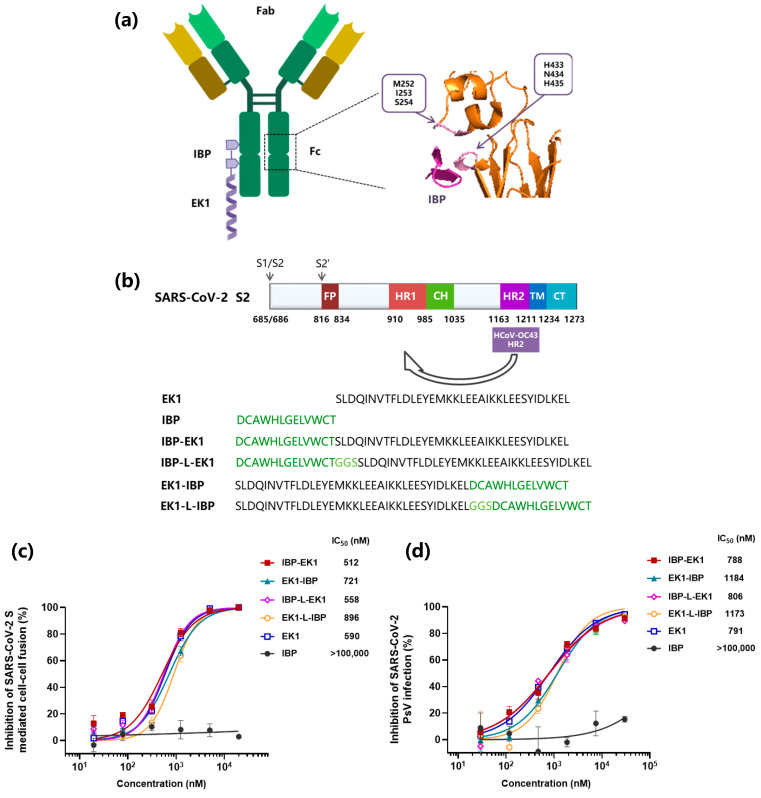
IBP conjugation as a strategy intended to prolong the half-life of HCoV peptide fusion inhibitors: (**a**) Schematic diagram of IBP-EK1 in complex with IgG Fc region and the interaction between IBP and Fc C_H_2/C_H_3 interface (PDB: 1DN2, presented by PyMOL). Critical residues of Fc C_H_2/C_H_3 interface binding to IBP are shown in boxes labeled pink on the interaction diagram and purple for IBP. C_H_2 and C_H_3 interact with IBP through M252, I253, and S254 and H433, N434, and H435, respectively. (**b**) Schematic diagram of HCoV S2 subunit composition (SARS-CoV-2, as an example), sequences of EK1 (derived from the HR2 region of HCoV-OC43), IBP, and conjugated peptides. FP, fusion peptide; HR1, heptad repeat 1; CH, central helix; HR2, heptad repeat 2; TM, transmembrane anchor; CT, cytoplasmic tail. (**c**) Inhibition of IBP-conjugated peptides on SARS-CoV-2 S-mediated cell–cell fusion. (**d**) Inhibitory activities of IBP-conjugated peptides against SARS-CoV-2 PsV infection. Each sample was tested in triplicate, and a representative example of three independent experiments was shown.

In the field of anti-HCoV agents, anti-CoV peptide fusion inhibitors can target conserved sites on the CoV S2 subunit to inhibit viral entry into cells, including 229E-HR2P derived from the HCoV-229E HR2 region [23], SARS-CoV fusion-inhibitory peptides derived from its HR2 region [24,25], and MERS-CoV HR2-derived peptide fusion inhibitors [26,27,28]. In previous studies, our team developed the first pan-CoV fusion inhibitory peptide, EK1 [29]. With origins in the HcoV-OC43 HR2 region (Figure 1a), EK1 targets the highly conserved heptad repeat 1 (HR1) region among HcoVs and thus blocks the formation of a six-helix bundle (6-HB) to effectively forestall virus entry into target cells. However, molecular weights of the above-mentioned peptide-based fusion inhibitors are usually small (<10 kDa), leading to their rapid clearance by glomerular filtration in the systemic circulation [30,31]. As a result, the in vivo half-lives of EK1 and similar peptide fusion inhibitors are short [32], needing frequent administration to maintain their required antiviral concentrations. This calls for the development of universal and long-acting strategies to slow down in vivo elimination and improve the bioavailability of CoV peptide-based fusion inhibitors.

With an inverse relationship between the renal clearance rate of protein-based biologics and their molecular weight [33], most long-acting strategies to reduce renal filtration and prolong half-life were achieved through increasing the (apparent) molecular weight of protein-based biologics. Currently, several marketed peptide drugs have adopted different strategies to optimize half-life, including conjugating polymers (e.g., polyethylene glycol (PEG) [34], fusion with long-circulating segments (hIgG Fc domain or human serum albumin (HSA) [35], or conjugating fatty acid chains (e.g., acylation)). These strategies have their own advantages but also shortcomings, as finally revealed by clinical application and research. Peptide PEGylation has contributed to issues like immunogenicity and safety [36,37,38], leading to adverse events and decreased therapeutic efficacy. Fusion with IgG Fc domain or albumin accounts for high molecular weight and thus increases steric hindrance, affects permeability, and reduces efficacy [39]. Accordingly, it is indispensable to develop novel long-acting strategies to improve the pharmacokinetics, especially the half-life, of CoV peptide fusion inhibitors in circulation, while also retaining their activity and safety.

In recent years, ligands in noncovalent combination with plasma proteins, including, for example, HSA and hIgG, have been extensively studied [40,41], and novel long-acting strategies for peptides based on these ligands have also attracted increasing attention. As a member of the immunoglobulin (Ig) superfamily, human immunoglobulin G (hIgG) is quite abundant in the serum, with a long half-life of up to 21 days [42], while conjugating IgG-binding ligands is less studied. DeLano et al. identified a 13-mer short peptide with high binding affinity to IgG Fc domain, termed Fc-III, after several rounds of in vitro screening of a bacteriophage display peptide library [43]. This peptide contains two cysteines (Cys), forming an internal disulfide bond, and thus displays as cyclic, specifically binding to the C_H_2 and C_H_3 interface of Fc domain. Considering the low molecular weight, simple structure, and nonbacterial origin of Fc-III [41], it is expected that conjugation of CoV peptide fusion inhibitors with Fc-III could retain their original advantages, including potent antiviral efficacy, safety profile, and low immunogenicity. Based on the abundance and the long half-life property of IgG in serum, conjugated peptides are expected to bind to IgG in vivo, thereby slowing down the elimination rate and extending the original half-life. Another promising therapeutic avenue is the application of conjugated peptides combined with monoclonal neutralizing antibodies, which are also IgG. Therefore, in this study, we used EK1 as a prototype model based on the IgG-binding peptide Fc-III and developed an Fc-binding strategy through conjugation with human immunoglobulin G Fc-binding peptide (IBP), or IBP-EK1, with the aim of generating a universal, long-acting, and broad-spectrum anti-CoV peptide fusion inhibitor.

We took several steps to realize this outcome. First, we designed IBP-conjugated peptides and detected their inhibitory activities, finally determining that IBP-EK1 had the best activity. Then, we demonstrated that IBP-EK1 showed stable inhibitory activity against all tested SARS-CoV-2 variants, especially the Omicron sublineages. IBP-EK1 also showed broadly inhibitory activity against other HCoVs in vitro, and it was effective in vivo to prevent and treat newborn mice challenged with HCoV-OC43. These results indicate that the strategy of IBP conjugation retained the original structure and inhibitory mechanism of EK1 but also acquired the feature of IBP’s intrinsic binding to IgG, significantly improving the proteolytic tolerance of EK1. After confirming the in vivo safety profile of IBP-EK1, we further evaluated the serum half-life (t_1/2_) of IBP-EK1 in rhesus monkeys, which was obviously prolonged compared with that of EK1. Meanwhile, we found the in vitro and ex vivo synergistic effects of IBP-EK1 when combined with monoclonal neutralizing antibodies (hIgG) targeting the receptor-binding domain (RBD) to inhibit sarbecovirus PsV, mainly SARS-CoV-2 variants. Collectively, these results suggest that IBP conjugation with CoV fusion inhibitory peptide is a promising long-acting strategy and that IBP-EK1 is a candidate for development as a long-acting and broad-spectrum antiviral agent to prevent and treat infection by SARS-CoV-2 variants, as well as other HCoVs, either alone or in combination with neutralizing antibodies.

## 2. Methods

### 2.1. Cell Lines, Viruses, Peptides, and Plasmids

Caco-2, Calu-3, RD, and 293T cells were obtained from American Type Culture Collection (ATCC; Manassas, VA, USA). Huh-7 cells were obtained from the Cell Bank of the Chinese Academy of Sciences (Shanghai, China). All cell lines were cultured in Dulbecco’s Modified Eagle’s Medium (DMEM) with 10% fetal bovine serum (FBS).

HCoV-OC43 (VR-1558) and HCoV-229E (VR-740) strains were obtained from ATCC and amplified in RD and Huh-7 cells, respectively. Authentic SARS-CoV-2 variant B.1.617.2 (Delta) was isolated from patients in Shanghai and preserved at the Biosafety Level 3 (BSL-3) Laboratory of Shanghai Medical College, Fudan University [44].

IBP and IBP-conjugated peptides (Figure 1b) were synthesized by Synpeptide Biotechnology Ltd. (Shanghai, China). EK1 (sequence: SLDQINVTFLDLEYEMKK LEEAIKKLEESYIDLKEL) [29] was synthesized by Chengdu Shengnuo Biotechnology Ltd. (Chengdu, China). SARS-CoV-2 HR1 peptide (HR1P, sequence: ANQFNSAIGKIQDSLSSTASALGKLQDVVNQNAQALNTLVKQ) was synthesized by GL Biochem Ltd. (Shanghai, China). All the peptides were tested by high-performance liquid chromatography with a purity of >95%.

Envelope-expressing plasmids, including pcDNA3.1-SARS-2-S, pcDNA3.1-SARS-2 variant-S, pcDNA3.1-SARS-S, pcDNA3.1-MERS-S, pcDNA3.1-WIV1-S, pcDNA3.1-OC43-S, pcDNA3.1-NL63-S, and pcDNA3.1-229E-S, and the luciferase reporter-expressing HIV-1 backbone (pNL4-3.Luc.R^−^.E^−^) were preserved in our laboratory [45]. The pAAV-S-IRES-EGFP fusion plasmids encoding EGFP and HCoV S protein, including pAAV-SARS-2-IRES-EGFP, pAAV-SARS-IRES-EGFP, pAAV-MERS-IRES-EGFP, pAAV-WIV1-IRES-EGFP, pAAV-OC43- IRES-EGFP, pAAV-NL63-IRES-EGFP, and pAAV-229E-IRES-EGFP, were constructed and preserved in our laboratory [45].

### 2.2. Inhibition of HCoV S-Mediated Cell–Cell Fusion

(1) The establishment and detection of cell–cell fusion assays mediated by S proteins of multiple HCoVs were as previously described [29,45]. In brief, 293T cells were transfected with the pAAV-S-IRES-EGFP fusion plasmids, as described in Section 2.1, to obtain 293T/S/EGFP cells, respectively, which were adopted as the effector cells. Caco-2 cells, expressing various HCoV receptors on the membrane surface, were used as target cells, as described below.

For SARS-CoV S-, WIV1 S-, OC43 S-, or NL63 S-mediated cell–cell fusion assays, effector cells and target cells were cocultured in DMEM containing trypsin (80 ng/mL) for 4 h, while for SARS-CoV-2 and MERS-CoV S-mediated cell–cell fusion assays, effector cells and target cells were cocultured in DMEM without trypsin for 2 h. After incubation, five fields in each well were randomly selected for counting the fused and unfused cells under an inverted fluorescence microscope (Invitrogen, Waltham, MA, USA). The percentage of cell–cell fusion ((number of fused cells/number of fused and unfused cells) × 100%) was then calculated.

(2) The inhibitory activity of a peptide on HCoV S-mediated cell–cell fusion was determined, as previously described [29,45]. Briefly, a total of 2.5 × 10^4^ cells/well target cells (Caco-2) were incubated for 12 h. Afterwards, 10^4^ cells/well effector cells (293T/S/EGFP) were added in the presence or absence of a peptide at the indicated concentrations at 37 °C for 2–4 h. After counting fused and unfused cells, the percentage of cell–cell fusion was calculated, as described above. The percent inhibition of cell–cell fusion was calculated using the following formula: (1 − E/P) × 100%, where “E” represents the percentage of cell–cell fusion in the peptide group, and “P” represents the percentage of cell–cell fusion in the positive control group, consisting of 293T/S/EGFP effector cells to which only DMEM was added. Samples were tested in triplicate, and all experiments were repeated twice.

### 2.3. Inhibition of Pseudotyped HCoV Infection

(1) To obtain various pseudotyped HCoVs, 293T cells were cotransfected with pNL4-3.Luc.R^−^.E^−^ and the envelope-expressing plasmids pcDNA3.1-S, as described in Section 2.1, respectively, using VigoFect (Vigorous Biotechnology, Beijing, China) [27]. Pseudotyped viral particles were efficiently released to the supernatant, which was harvested at 48 h post transfection, filtrated through a 0.45-μm filter (PALL, New York, NY, USA) and frozen at −80 °C.

(2) To detect the inhibitory activity of a peptide on infection of pseudotyped HCoV, target cells (Caco-2 for all tested CoVs, 0.7 × 10^4^ cells/well; Calu-3 for SARS-CoV-2 and its variants, MERS-CoV, SARS-CoV, and HCoV-NL63, 1.5 × 10^4^ cells/well) were plated at an appropriate density per well in a 96-well plate 24 h prior to infection. Pseudoviruses were mixed with an equal volume of serially diluted peptide solutions (with non-FBS DMEM), and the mixtures were incubated at 37 °C for 40 min, followed by transfer to the 96-well plate with target cells. Supernatants were replaced after 10~12 h and incubated for another 48 h. Luciferase activities were determined by the Luciferase Assay System (Promega, Madison, WI, USA) and detected by the Multimode Reader (Tecan, Maennedorf, Switzerland) [27].

### 2.4. Inhibition of Live HCoV Replication

(1) The inhibition assay for live SARS-CoV-2 Delta variant was performed in the Biosafety Level 3 (BSL-3) Laboratory in Shanghai Medical College of Fudan University, as described previously [32]. Serially diluted peptide solutions were mixed with an equal volume of live SARS-CoV-2 Delta variant (100 TCID_50_), incubated at 37 °C for 30 min, and then added to precultured Calu-3 cells. After 2 h of incubation at 37 °C, the supernatant was replaced with DMEM containing 2% FBS. After incubation for an additional 48 h, the supernatants were harvested and collected in freezing tubes containing TRIzol™ LS Reagent (Invitrogen, Waltham, MA, USA), followed by storage at −80 °C. The Total RNA Extraction Kit (Tiangen Biochem Ltd., Beijing, China) was used to extract RNA from the supernatant samples. Real-time quantitative PCR (RT-qPCR) assay was performed to determine RNA copies by using the N gene probe and One Step PrimeScript^TM^ RT-PCR Kit (Takara, Shiga, Japan).

The probe and primers for detecting SARS-CoV-2 N gene in RNA samples were as follows: Forward primer: 5′ GGGGAACTTCTCCTGCTAGAAT 3′; Reverse primer: 5′ CAGACATTTTGCTCTCAAGCTG 3′; and N gene probe: 5′ FAM-TTGCTGCTGCTT GACAGATT-TAMRA 3′.

(2) The inhibitory activity of the tested peptide against HCoV-OC43 replication in RD cells or HCoV-229E replication in Huh-7 cells was evaluated [45]. Briefly, 60 μL of HCoVs (100 TCID_50_) was mixed with an equal volume of serially diluted peptide solutions and incubated at 37 °C for 40 min. Afterwards, the mixtures were added to RD or Huh-7 cells in a 96-well microplate. Supernatants were replaced after 6~8 h and incubated for an additional 48 h. The cytopathic effect caused by HCoV infection was determined by Cell Counting Kit-8 (CCK8; Dojindo, Japan) assay and measured by the Multi-Detection Microplate Reader (Tecan, Maennedorf, Switzerland) at a wavelength of 450 nm (OD_450_).

### 2.5. In Vivo Protective Study against Live HCoV-OC43 Infection

Newborn mice were bred from pregnant ICR mice (17 days) purchased from Slac Laboratory Animal Ltd. (Shanghai, China). All related experiments were carried out in strict accordance with institutional regulations. Ten suckling mice (3-day-old) were allocated randomly to each group. Mice in the prophylactic and therapeutic groups were intranasally administered with peptide (10 mg/kg in 4 μL of PBS) 1 h before or after intranasal challenge with 100 TCID_50_ HCoV-OC43 (in 4 μL PBS). In detail, 1 h before (for testing prophylactic effect) or 1 h after (for testing therapeutic effect) HCoV-OC43 challenge, a single dose of 10 mg/kg IBP-EK1 or EK1 (in 4 μL PBS) was given to newborn mice by alternately dropping into their bilateral nostrils through a metallic syringe with a blunt needle. HCoV-OC43 challenge was carried out in a biosafety cabinet, where 4 μL of HCoV-OC43 diluent (with a viral load of 100 TCID_50_) was slowly dropped into a unilateral nostril of each mouse through a metallic syringe with a blunt needle. Mice in the viral control group were intranasally administered with 4 μL of PBS 1 h before or after viral challenge.

In each group, five mice were randomly selected for euthanasia on day 5 after challenge, among which four mice were assessed for viral titer in mouse brain, as described below, and one mouse was assessed for brain histopathologic examination [46]. The survival and body weights of the remaining mice in each group were monitored for the subsequent 14 days.

Primers for detection of viral RNA were as follows: HCoV-OC43 S: Forward primer 5′ GACACCGGTCCTCCTCCTAT 3′; Reverse primer 5′ ACACTTCCCTTC AGTGCCAT 3′. GAPDH: Forward primer 5′ TGCTGTCCCTGTATGCCTCTG 3′; Reverse primer 5′ TTGATGT CACGCACGATTTCC 3′.

### 2.6. Circular Dichroism Spectroscopic Analysis

As previously described [45], individual peptides were dissolved in phosphate buffer to prepare a solution with a final concentration of 10 μM. Peptide mixtures were also dissolved in phosphate buffer to 10 μM, respectively, and incubated at 25 °C for 40 min. Their circular dichroism spectra (scanning wavelength range: 200 to 260 nm) were measured on a J-815 spectrometer (JASCO Inc., Tokyo, Japan). To evaluate the thermostability of peptide complexes, thermal denaturation was monitored from 20 to 98 °C at 222 nm with a thermal gradient of 5 °C/min. The midpoint of melting curve, or Tm value, was acquired by JASCO software (J-815) utilities.

### 2.7. Expression and Purification of IgG/Antibody

(1) To express and purify neutralizing antibody, including S309 and 10933, Expi293 cells were cultured in suspension using SMM 293-TII expression medium (Sino Biological Inc., Beijing, China) at 37 °C in a 5% CO_2_ incubator rotating at 120 rpm. With a density of 1 × 10^6^ cells/mL, the cultures per 50 mL were cotransfected with 30 µg of heavy chain and 30 µg of light chain-encoding plasmids using 180 µL EZ Trans (Life iLab Biotech, Shanghai, China) and cultured for 6 days. Then, cells and cellular debris were removed by centrifugation at 3000× *g* rpm, 4 °C for 25 min, followed by filtration through a 0.45 µm filter. Clarified cell supernatant containing antibodies was shaken with and thus bound to the equilibrated rProtein G resin (Smart-Lifesciences Biotechnology Ltd., Changzhou, China) at 4 °C for about 8 h. The resin was extensively washed with PBS and eluted with IgG elution buffer (0.1 M Glycine, pH 2.9) and neutralization buffer (1 M Tris-HCl, pH 9.0). Purified S309 or 10,933 was ultrafiltrated, concentrated in PBS, and stored at −80 °C.

(2) To purify rhesus monkey IgG from sera, 1 mL of rhesus monkey serum was diluted 50-fold with PBS and filtrated with a 0.45 μm filter, and the equilibrated rProtein G resin was added to the serum diluent. After shaking and binding at 4 °C for about 6 h, the resin was extensively washed with PBS, eluted with IgG elution buffer, and neutralized, as described above. Purified rhesus monkey IgG was stored at 4 °C or −80 °C for a long period.

### 2.8. Measurement of Peptide Binding to IgG

(1) The binding abilities of peptides to human immunoglobulin G (IgG) (Thermo Fisher Scientific, Waltham, MA, USA) or rhesus monkey IgG (purified from sera of rhesus monkeys) were evaluated by enzyme-linked immunosorbent assay (ELISA), as described elsewhere [47]. A 5 μg/mL solution of IBP, EK1, and IBP-EK1 was coated on wells of a 96-well ELISA plate at 4 °C overnight. After blockage for 2 h at 37 °C, serially diluted solutions of human (or rhesus monkey) IgG were added to the peptide-coated plate, incubated at 37 °C for 1 h, and washed 3 times with PBS containing 0.05% Tween-20 (0.05% PBST). Then, goat antihuman IgG-HRP (1:4000 dilution) was added to each well, incubated at 37 °C for another 1 h, and washed 5 times. Afterwards, the bound IgG-HRP was detected by addition of HRP substrate TMB, and the reaction was terminated by H_2_SO_4_ (1 M). Absorbance at 450 nm (OD_450_) was measured using the Multimode Reader (Tecan, Maennedorf, Switzerland). Each sample was tested in triplicate, and data are presented as means ± SD.

(2) The binding affinities of peptides to human IgG (Thermo Fisher Scientific) or rhesus monkey IgG (purified from sera of rhesus monkeys, as described in 2.7) were determined by biolayer interferometry (BLI). IgG was diluted with 0.02% PBST to a concentration of 10 μg/mL, and 5 μM of peptide was 4-fold serially diluted with 0.02% PBST. AHC biosensors (Forte Biosciences Inc., Dallas, TX, USA) were hydrated in ddH_2_O and equilibrated in 0.02% PBST for 300 s, and IgG (10 μg/mL) was loaded to the biosensors. The sensors were then immersed in graded peptide solutions (0–5 μM) for 300 s prior to dissociation in 0.02% PBST buffer for another 300 s at 30 °C and 1000 rpm with shaking on an Octet RED instrument (Forte Biosciences Inc., Dallas, TX, USA) to measure the association rate constants (Kon) and dissociation rate constants (Koff) for the complex between peptide and IgG. Global curve fitting was performed using a 1:1 binding model and the ForteBio software (Data Analysis 8.1) to obtain the equilibrium dissociation constant (K_D_). All experiments were repeated twice, and the result shown was representative of all.

### 2.9. Evaluation of Tolerance to Proteolytic Enzymes

Assays for the stability of peptides to proteolytic enzymes, including proteinase K and trypsin, were performed as previously described [44]. For trypsin tests, 10 μM peptide was incubated with 25 μg/mL trypsin (Sigma-Aldrich, Burlington, MA, USA) at 37 °C, and samples under different incubation times (0, 10, 30, 60, 120, 240, and 360 min) were collected. The sample was immediately added with FBS (10% *v*/*v*) to neutralize the proteolytic activity of trypsin, followed by inactivating trypsin at 56 °C for 30 min. Then, supernatants were absorbed and stored at −80 °C before testing. For proteinase K tests, 10 μM peptide was incubated with 100 μg/mL proteinase K (Sigma-Aldrich) at 37 °C, and samples under different incubation times (0, 10, 30, 60, 120, 240, and 420 min) were collected and centrifuged (10,000× *g* rpm, 2 min, 4 °C) immediately. The supernatant was absorbed and stored at −80 °C before testing. The residual antiviral activity of each sample was detected by PsV inhibition assay as described above.

### 2.10. Cytotoxicity Assay

Cytotoxicity of peptides to the target cells (Caco-2, Calu-3, RD and Huh-7 cells) was tested by using the Cell Counting Kit-8 (CCK-8; Dojindo, Kumamoto, Japan). Briefly, each cell type was seeded into wells of a 96-well microplate (Caco-2: 0.7 × 10^4^ cells/well; Calu-3: 1.5 × 10^4^ cells/well; RD and Huh-7: 10^4^ cells/well) and cultured at 37 °C. After incubation for 12 or 24 h, serially diluted peptides were added to the wells. After subsequent incubation at 37 °C for 48 h, CCK-8 solution (1:20 dilution, 100 μL/well) was added, followed by an additional incubation for about 3 h, after which OD_450_ was measured.

### 2.11. In Vivo Safety Assay

As previously described, female ICR mice (7-week-old) were assigned randomly to three groups (*n* = 3), respectively, administered with PBS, 50 mg/kg IBP-EK1 intranasally and 50 mg/kg IBP-EK1 intraperitoneally every day for 5 days. Bodyweight changes were monitored over the following 14 days. Alanine aminotransferase (ALT) and creatinine (CRE) in the sera of each group were measured using the ALT and creatinine assay kits (NJJCBIO, Nanjing, China) before the first administration and 1, 3, and 5 days after the final administration. The titer of antibodies to IBP-EK1 in the sera of each group was evaluated by ELISA 14 days after the final administration. Twenty-eight days after administration, mice in each group were euthanized to harvest the lungs, livers, and kidneys for hematoxylin and eosin staining.

### 2.12. Pharmacokinetic Studies in Rhesus Monkeys

(1) Rhesus monkeys were raised at Beijing Vafory Biotechnology Co., Ltd., which also performed peptide administration and blood collection. Briefly, rhesus monkeys were administered intravenously with a single dose of 10 mg/kg IBP-EK1 (*n* = 2) or EK1 (*n* = 2). Serial blood samples were collected from monkeys that received IBP-EK1 or EK1 before injection and at 2, 4, 6, 24, 72, and 144 h post injection. The samples were then centrifuged at 3000× *g* rpm, 4 °C for 10 min, and sera were aliquoted and kept frozen until analysis.

(2) As described previously, concentrations of the active peptide in sera can be estimated by detecting the ex vivo antiviral activities of the treated sera [48,49]. Briefly, the ex vivo anti-HCoV (SARS-CoV, as an example) activities of IBP-EK1-treated or EK1-treated sera from rhesus monkeys were first detected. Then, dilution folds of sera causing 50% inhibition of SARS-CoV PsV infection (DF-IC_50_s) were calculated. Based on the in vitro IC_50_ against SARS-CoV PsV and DF-IC_50_ values calculated herein, concentrations of IBP-EK1 or EK1 in the sera were estimated, and their half-lives and other pharmacokinetic parameters were calculated using MODFIT software (V6.5), as previously described [50].

(3) High-performance liquid chromatography–tandem mass spectrometry (HPLC-MS/MS) can be used to accurately determine the concentrations of IBP-EK1 or EK1 in sera and their half-lives. Briefly, we generated a standard curve of IBP-EK1 or EK1, respectively. To accomplish this, a working solution of 10 μg/mL IBP-EK1 (or EK1) was prepared with methanol, and standard solutions of 2500, 1000, 500, 250, 100, and 50 ng/mL peptide were prepared, respectively, with 50% acetonitrile solution. The standard solutions were then sent into ultraperformance liquid chromatography–quadrupole time-of-flight mass spectrometry (UPLC-Q-TOF-MS; SCIEX Inc., Framingham, MA, USA) for detection with the mobile phase A of liquid chromatography being pure water and mobile phase B being acetonitrile containing 0.1% formic acid. Afterwards, we measured the IBP-EK1-treated (or EK1-treated) sera from rhesus monkeys. Serum samples were diluted 10-fold and injected into UPLC-Q-TOF-MS for detection. According to its standard curve, IBP-EK1 (or EK1) concentrations in serum samples at different time points after administration were calculated. Then, the serum half-life and other pharmacokinetic parameters of IBP-EK1 (or EK1) were fit by MODFIT software (V6.5), as described above.

### 2.13. Detection of Synergistic Antiviral Effect of IBP-EK1 in Combination with Neutralizing Antibodies

To evaluate the potential synergistic effect, neutralizing antibody and IBP-EK1 were mixed at the indicated molar concentration ratio, whereas neutralizing antibody alone and IBP-EK1 alone were also included as controls. The samples were serially diluted and tested for their inhibitory activities on SARS-CoV-2 PsV infection, as described in Section 2.11. The data were analyzed for synergistic effect by calculating the combination index (CI) with CalcuSyn software (Copyright 2006), kindly provided by Dr. T. C. Chou [51,52]. As described previously, CI values of <0.95 and >1.05 indicate synergism and antagonism, respectively. CI values of <0.1, 0.1–0.3, 0.3–0.7, 0.7–0.85, and 0.85–0.90 indicate very strong synergism, strong synergism, synergism, moderate synergism, and slight synergism, respectively [51]. The fold of dose reduction was calculated according to concentrations of inhibitors tested alone and in combination.

### 2.14. Detection of Ex Vivo Synergistic Effect of IBP-EK1 in Combination with Neutralizing Antibodies

The ex vivo anti-HCoV (SARS-CoV-2, as an example) activities of mice sera treated with the peptide or neutralizing antibody or their combination were detected, as described previously [47,53]. Briefly, female BALB/c mice (8-week-old) were intraperitoneally administered with 20 mg/kg S309, 5 mg/kg S309, 15 mg/kg IBP-EK1, S309 (5 mg/kg)/IBP-EK1 (15 mg/kg) in combination, or PBS (as a control), respectively. Sera were collected from these mice at 2 and 8 h post injection and tested for their inhibitory activities against SARS-CoV-2 PsV infection, as described above, and dilution folds of sera causing 50% inhibition (DF-IC_50_) were calculated to determine the ex vivo synergistic effect of IBP-EK1 in combination with S309.

### 2.15. Statistical Analyses

Statistical analyses were carried out using GraphPad Prism 8.0, and the IC_50_ values were calculated using the CalcuSyn software (Copyright 2006) provided by T. C. Chou [51]. Statistical differences were analyzed by unpaired two-tailed *t*-test and also with GraphPad Prism 8.0. *p* > 0.05 was considered not statistically significant, and *p* < 0.05 was considered statistically significant (* *p* < 0.05, ** *p* < 0.01, *** *p* < 0.001, and **** *p* < 0.0001).

## 3. Results

### 3.1. Design and Identification of IBP-Conjugated Peptides

Several short peptides were reported to specifically bind to human IgG [41]. Among them, IBP (Fc-III) showed explicit binding sites and relatively high binding affinity, mainly to the C_H_2 and C_H_3 interface of the human IgG Fc region (Figure 1a) [43]. To take advantage of this IBP-specific property, we designed and synthesized four peptides by conjugating IBP to the N- or C-terminus of EK1 with or without a linker (sequence: GGS) between IBP and EK1, i.e., IBP-EK1, EK1-IBP, IBP-L-EK1, and EK1-L-IBP, respectively (Figure 1b).

The widely used HCoV S protein-mediated cell–cell fusion model can be used to assess the inhibitory activity of HCoV fusion inhibitors [54]. Here, we systematically assessed the inhibitory activities of IBP-conjugated peptides on SARS-CoV-2 S protein-mediated cell–cell fusion system, using IBP as the negative control and EK1 as the positive control. As the results show in Figure 1c, IBP-EK1 displayed more efficacy in inhibiting cell–cell fusion (IC_50_: 512 nM) than EK1-IBP (IC_50_: 721 nM), EK1-L-IBP (IC_50_: 896 nM), IBP-L-EK1 (IC_50_: 558 nM), and EK1 (IC_50_: 590 nM). Consistently, IBP-EK1 also showed the most potent inhibitory activity against SARS-CoV-2 pseudovirus (PsV) infection (Figure 1d). Therefore, we selected IBP-EK1 to perform subsequent investigations.

### 3.2. IBP-EK1 Showed Potent Inhibitory Activities against SARS-CoV-2 Variants

Inhibitory activities of most neutralizing antibodies against SARS-CoV-2 variants, especially the Omicron sublineages, were reported to decrease [22]. To investigate whether multiple mutations in the spike proteins of SARS-CoV-2 variants could affect the inhibitory activities of IBP-EK1, we tested its efficacy on a variety of SARS-CoV-2 pseudotyped variants.

**Figure 2:**
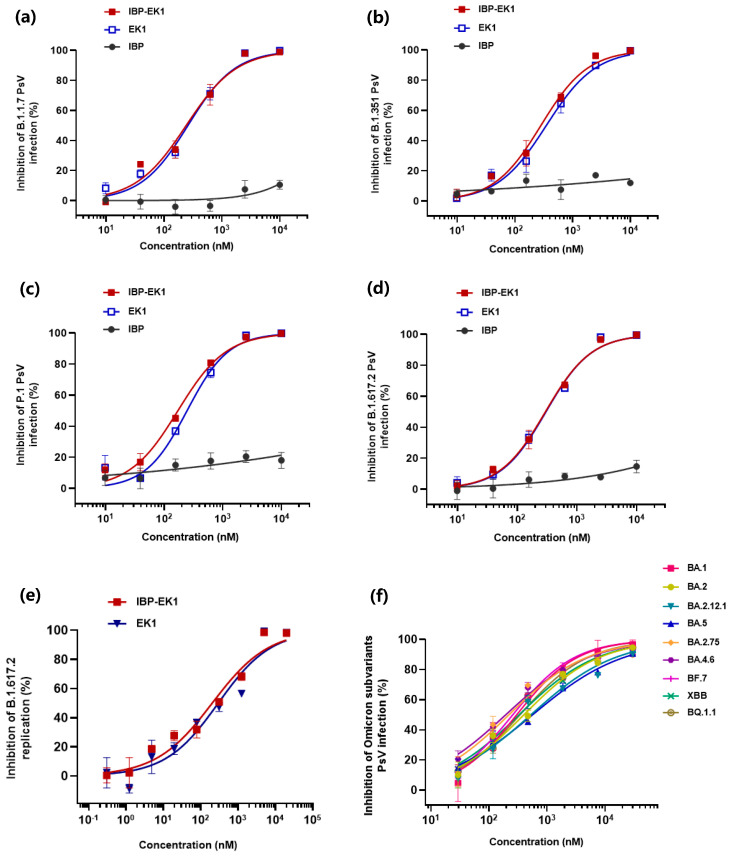
Potent inhibitory activities of IBP-EK1 against pseudotyped SARS-CoV-2 variants infection: (**a**–**d**) Inhibitory activities of IBP-EK1 and EK1 in PsV infection assays on Calu-3 cells against SARS-CoV-2 previous variants of concern B.1.1.7 (Alpha) (**a**), B.1.351 (Beta) (**b**), P.1 (Gamma) (**c**), and B.1.617.2 (Delta) (**d**). (**e**) Inhibitory activities of IBP-EK1 and EK1 in live SARS-CoV-2 infection assays on Calu-3 cells against B.1.617.2 (Delta) variant. (**f**) Inhibitory activities of IBP-EK1 in PsV infection assays on Caco-2 cells against the Omicron subvariants. Each sample was tested in triplicate, and a representative example of three independent experiments is shown.

As shown in Figure 2a–d and Table 1, IBP-EK1 could effectively inhibit the infection of pseudotyped SARS-CoV-2 VOCs on Calu-3 cells, including B.1.1.7 (Alpha), B.1.351 (Beta), P.1 (Gamma), and B.1.617.2 (Delta), with IC_50_ values ranging from 171 to 296 nM, similar to EK1. Similar results were observed on Caco-2 cells, with IBP-EK1 showing IC_50_ values that ranged from 338 to 722 nM (Figure S1 and Table 1). As Omicron has more mutation sites in SARS-CoV-2 S2 subunit than previous variants, we focused on the inhibitory activities of IBP-EK1 against Omicron sublineages. As shown in Figure 2f, IBP-EK1 was not impacted by increasing mutations of Omicron sublineages, especially the recently prevalent BA.5, BF.7, BQ.1.1, and XBB, with IC_50_ values ranging from 195 to 564 nM. Therefore, the results demonstrate that IBP-EK1 exhibited effective and stable inhibitory activities against all tested SARS-CoV-2 pseudotyped variants with IC_50_s in the range of 19~722 nM.

We further tested the inhibitory effect of IBP-EK1 on authentic SARS-CoV-2 (represented by Delta variant) proliferation. Through one-step real-time quantitative PCR (RT-qPCR) assay, absolute quantification of viral RNA copies in different samples was carried out according to their Ct values, and, thus, the inhibition of IBP-EK1 at different concentrations against the Delta variant was calculated. As shown in Figure 2e, IBP-EK1 could block the proliferation of live Delta variant in a dose-dependent manner with an IC_50_ value of 204 nM. All these results indicate that IBP-EK1 exhibited broadly inhibitory activities against SARS-CoV-2 and its variants.

### 3.3. IBP-EK1 Exhibited Broadly Inhibitory Activities against HCoVs In Vitro

**Figure 3:**
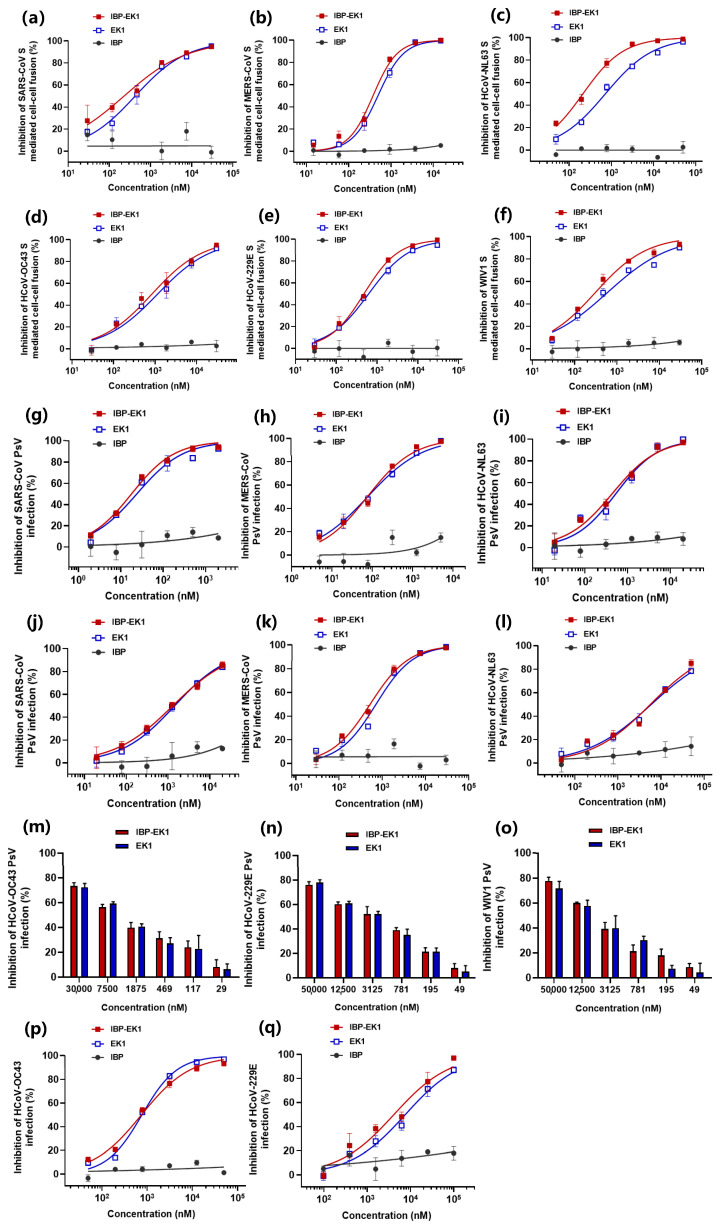
Broadly inhibitory activities of IBP-EK1 against cell–cell fusion mediated by S protein and PsV infection of different genera of CoVs and live HCoV replication: (**a**–**f**) Inhibitory activities of IBP-EK1 and EK1 in cell–cell fusion mediated by the S proteins of SARS-CoV (**a**), MERS-CoV (**b**), HCoV-NL63 (**c**), HCoV-OC43 (**d**), HCoV-229E (**e**), and WIV1-CoV (**f**). (**g**–**l**) Inhibitory activities of IBP-EK1 and EK1 in PsV infection assays on Caco-2 cells against SARS-CoV (**g**), MERS-CoV (**h**), and HCoV-NL63 (**i**) and on Calu-3 cells against SARS-CoV (**j**), MERS-CoV (**k**), and HCoV-NL63 (**l**). (**m**–**o**) Inhibitory activities of IBP-EK1 and EK1 in PsV infection assays against HCoV-229E (**m**), HCoV-OC43 (**n**), and WIV1-CoV (**o**). (**p**,**q**) Inhibitory activities of IBP-EK1 and EK1 on live HCoV replication for HCoV-229E (**p**) and HCoV-OC43 (**q**). Each sample was tested in triplicate, and a representative example of three independent experiments is shown.

To further investigate the breadth and potency of IBP-EK1 against other HCoVs, we first evaluated the effect of peptides on CoV S protein-mediated cell–cell fusion. Although some CoVs are not identified as HCoVs, they have been proven to be infectious to human cells. Therefore, we chose WIV1-CoV, belonging to SL-CoVs [12,13], to evaluate together with HCoVs. As shown in Figure 3a–f and Table 2, S protein-mediated cell–cell fusion of SARS-CoV, WIV1-CoV, MERS-CoV, and HCoV-OC43, belonging to β-CoVs, could be effectively blocked by IBP-EK1 with IC_50_ values ranging from 229 to 829 nM, which is more potent than EK1. For HCoV-NL63 and HCoV-229E, belonging to α-CoVs, IBP-EK1 also exerted strong inhibitory effects on their cell–cell fusion with IC_50_ values of 212 and 478 nM, respectively, which is also stronger than EK1.

We then evaluated the effect of peptides on PsV infection of the CoVs described above. As shown in Figure 3j–o and Table 2, IBP-EK1 could also effectively inhibit the infection of pseudotyped CoVs on Caco-2 cells with IC_50_ values ranging from 522 to 6047 nM. Interestingly, as demonstrated in Figure 3g–i, against the same HCoVs, IBP-EK1 showed significantly stronger inhibitory activities on Calu-3 cells than those on Caco-2 cells with IC_50_ values against SARS-CoV, MERS-CoV, and HCoV-NL63 PsV of 18, 76, and 440 nM, respectively. Further, we tested the in vitro inhibitory activities of IBP-EK1 against live HCoV infection. We amplified HCoVs with obvious CPE effects on target cells, including HCoV-OC43 and HCoV-229E. The inhibitory effects of IBP-EK1 on these two HCoVs were measured by CCK8 assay with IC_50_ values of 744 nM and 4139 nM, respectively (Figure 3p,q and Table 2). Hence, consistent with results of cell–cell fusion and PsV infection, IBP-EK1 inhibited the proliferation of live HCoVs efficiently. All IC_50_s of IBP alone exceeded 50 μM, showing no obvious inhibitory activity in any cell–cell fusion, PsV, or live virus infection experiments.

For a variety of CoVs, these results show that IBP-EK1 exhibited broad and potent in vitro inhibitory activities (Table 2).

### 3.4. Intranasal Administration of IBP-EK1 Strongly Protected Mice against HCoV-OC43 Challenge

In order to further investigate the clinical potential of IBP-EK1, we studied the in vivo protective effect of IBP-EK1 against HCoV infection. It has been reported that HCoV-OC43 can infect susceptible mice with viral attack on neurons, causing chronic encephalitis, disability, and even death [55]. Therefore, we evaluated the in vivo prophylactic and therapeutic effects of IBP-EK1 through challenging newborn mice with live HCoV-OC43.

**Figure 4:**
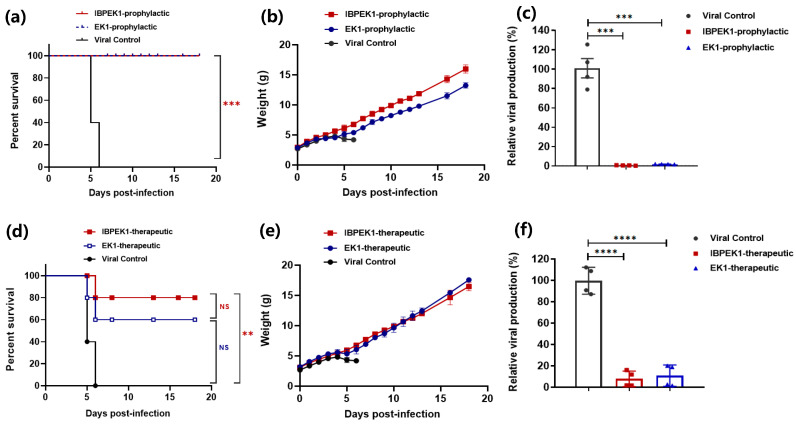
In vivo prophylactic and therapeutic efficacy of IBP-EK1 against HCoV-OC43 infection in newborn mice: (**a**) Survival curves of 3-day-old suckling mice administered with 10 mg/kg IBP-EK1 or EK1 1 h before HCoV-OC43 (100 TCID_50_) challenge. (**b**) Body weight change in each group. (**c**) Relative viral production in mouse brain of each group, calculated as 2^−ΔΔCt^. Unpaired, 2-tailed *t*-test was performed. *** *p* < 0.001. (**d**) Survival curves of 3-day-old suckling mice treated with 10 mg/kg IBP-EK1 or EK1 1 h after HCoV-OC43 (100 TCID_50_) challenge. (**e**) Body weight change in each group. (**f**) Relative viral production in mouse brain of each group, calculated as 2^−ΔΔCt^. Unpaired, 2-tailed *t*-test was performed. No significance (NS), *p* > 0.05; ** *p* < 0.01, and **** *p* < 0.0001. A representative example of two independent experiments is shown.

First, we studied the prophylactic effect of IBP-EK1 against HCoV-OC43 infection in newborn mice. Suckling mice were administered intranasally with a single dose of 10 mg/kg IBP-EK1 or EK1, followed by challenge with HCoV-OC43 (100 TCID_50_) one hour later. As demonstrated in Figure 4a,b, mice in the viral control group exhibited twitching limbs, unsteadiness, weight loss, and even death. Mortality reached 100% on the 6th day after challenge. For the IBP-EK1 or EK1 prophylactic group, 100% protection was achieved (Figure 4a). On the 5th day post challenge, the brain tissues of mice were separated to extract RNA, and relative quantification was carried out. Compared with the viral control group, results show that viral loads of the IBP-EK1 or EK1 prophylactic group were very low, with a statistically significant difference (Figure 4c).

We then evaluated the therapeutic effect of IBP-EK1 against HCoV-OC43 infection in newborn mice. One hour after HCoV-OC43 challenge (100 TCID_50_), suckling mice were given 10 mg/kg IBP-EK1 or EK1 via the intranasal route, and their survival rates and weights were monitored in the following days. Mice in the viral control group showed obvious weight loss 4 days after challenge, and their mortality reached 100% 6 days after challenge. In contrast, mice in IBP-EK1 therapeutic group achieved 80% protection, significantly different from that of the viral control group (Figure 4d,e). Consistently, viral loads of the IBP-EK1 or EK1 therapeutic group were significantly different from those of viral control group, suggesting that HCoV-OC43 infection could be effectively inhibited (Figure 4f).

In addition, the brain tissues of mice were analyzed from prepared sections, and H&E staining was performed. In contrast to brain tissues of the viral control group with such structural abnormalities as degeneration, vacuolation, and inflammatory infiltration, no apparent histopathological changes were observed in either the IBP-EK1 prophylactic or therapeutic group (Figure S2), further suggesting that IBP-EK1 affords potent in vivo efficacy to prevent and treat HCoV infection.

### 3.5. Influences of IBP Conjugation on the Original Properties of Peptides

We confirmed that IBP-EK1 exhibited inhibitory activities as broad and potent as those of EK1 in vitro and in vivo. Further, by means of circular dichroism (CD) and biolayer interferometry (BLI), we explored the effects of IBP conjugation on the original properties and functions of peptides.

**Figure 5:**
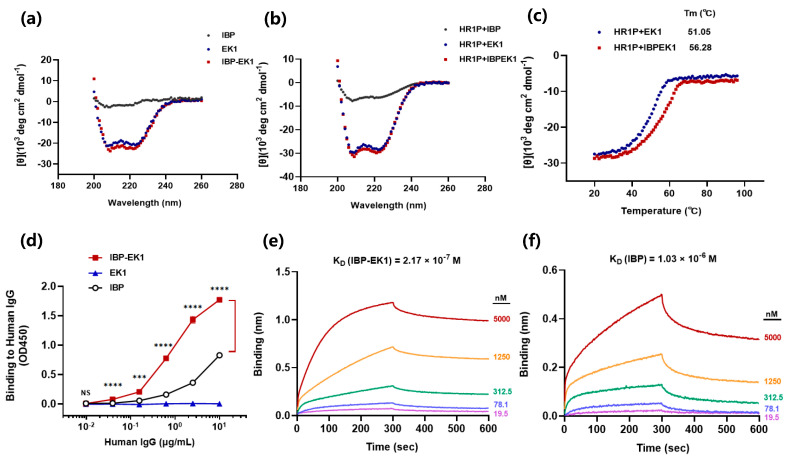
Biophysical properties of IBP-EK1: (**a**) Circular dichroism (CD) spectra for IBP, EK1, and IBP-EK1 in phosphate buffer (pH = 7.2). (**b**) CD spectra of the secondary structure of SARS-CoV-2 HR1P in complex with IBP, EK1, or IBP-EK1 in phosphate buffer (pH = 7.2). EK1 and IBP were used as positive and negative controls, respectively. CD spectrum displayed typical double minima at 208 and 222 nm for the α-helical feature. (**c**) Thermostability of SARS-CoV-2 HR1P in complex with EK1 or IBP-EK1 in phosphate buffer (pH = 7.2). (**d**–**f**) Binding of IBP, EK1, and IBP-EK1 to human IgG, as detected by ELISA and BLI. NS, *p* > 0.05; *** *p* < 0.001, and **** *p* < 0.0001. In the BLI assay, 10 μg/mL human IgG was immobilized at the surface of Fc biosensors, and then diluted peptide solutions were loaded. A representative example of two independent experiments is shown. Data were analyzed and globally fit with Data Analysis (V8.1). Equilibrium dissociation constants (K_D_) are reported above the plot.

To explore whether IBP conjugation could affect the structure of EK1, we determined the secondary structure of IBP-EK1 through CD spectra. As shown in Figure 5a, IBP-EK1 displayed double negative peaks at 208 and 222 nm, typical of α-helix in the CD spectrum and similar to that of EK1. As expected, IBP itself had no α-helical feature. This result demonstrates that the two peptides had similar α-helical characteristics, indicating that IBP conjugation to N-terminus of EK1 did not change its original conformation.

Next, we continued to determine whether IBP conjugation would affect the mechanism of action of EK1. For many type I enveloped viruses, including SARS-CoV-2, it is well known that the formation of the 6-HB fusion core is a key step for fusion of viral envelope with the target cell membrane and viral entry. As mentioned above, EK1, which is derived from the HR2 region of HCoV-OC43, interacts with the HR1 region in the fusion process and thus blocks the formation of 6-HB between HR1 and HR2 trimer. With EK1 as the positive control, IBP-EK1 was incubated with an equal molar concentration of HR1P (a peptide derived from the HR1 region; SARS-CoV-2 HR1P, as an example) at 25 °C for 40 min, and CD spectra of EK1/HR1P and IBP-EK1/HR1P mixtures were measured, respectively. As shown in Figure 5b, the EK1/HR1P mixture exhibited significantly deeper double negative peaks at 208 and 222 nm, typical of 6-HB in the CD spectrum, indicating that the interaction between EK1 and HR1P formed a complex with high α-helix content, i.e., heterogeneous 6-HB. Compared with IBP-EK1 alone, the IBP-EK1/HR1P mixture exhibited significantly deeper double negative peaks, similar to those of the EK1/HR1P mixture. These results indicate that like EK1 and other HR2P peptides, IBP-EK1 can also interact with HR1P to form heterogeneous 6-HB, suggesting that IBP-EK1 adopts the same mechanism of action as EK1 by targeting the conserved HR1 region to combat CoV infection.

To further study thermostability of the complex formed by EK1 or IBP-EK1 with HR1P, we detected the changes in their CD spectra at 222 nm as temperature increased. As shown in Figure 5c, with the temperature increasing gradually, the signal of CD was weakened, indicating that 6-HB structures of the complexes were gradually denatured and dissociated, eventually tending toward a relatively stable value. Then, the midpoint temperatures of melting curves, namely melting temperatures (Tm), were obtained through the analysis software in the instrument. Results show that the 6-HB structure formed between IBP-EK1 and HR1P had higher Tm, indicating better thermostability. These results suggest that IBP-EK1 may inhibit HCoV infection more efficiently than EK1 by forming a more stable heterogeneous 6-HB with the HR1 trimer.

Previous studies have shown that IBP can specifically bind to the Fc domain of human IgG. To investigate whether IBP conjugation with EK1 would affect the binding of IBP to IgG Fc, we detected the binding of IBP-EK1 to human IgG by ELISA. As shown in Figure 5d, the absorbance (OD_450_) of IBP-EK1 at 450 nm was positively correlated with the concentration of human IgG, showing that IBP-EK1 could indeed bind to human IgG and significantly more strongly than IBP itself. In order to clarify the binding of IBP-EK1 to IgG more directly, BLI was used to determine the binding affinity of IBP-EK1 with human IgG. The Kon and Koff between IBP-EK1 or IBP and human IgG were measured, and their ratio (Koff/Kon) is the equilibrium dissociation constant K_D_ (Table S3). As shown in Figure 5e,f, IBP-EK1 displayed higher binding affinity to hIgG (K_D_ = 2.17 × 10^−7^ M) than that of IBP itself (K_D_ = 1.03 × 10^−6^ M), being about 3.6-fold more potent.

It has been reported that high homology exists between rhesus monkey and the human IgG Fc domain [56,57]. Through sequence alignment, we found that IBP-binding sites (residues 252–254 and 433–435) at C_H_2 and C_H_3 interface of human IgG Fc were identical to those of rhesus monkey IgG (Figure S3a). Therefore, we also detected the binding of IBP-EK1 to rhesus monkey IgG. As shown in Figure S4 and Table S3, the binding of IBP-EK1 to monkey IgG was also obviously enhanced compared with that of IBP alone. In sum, these collective results show that the conjugation between IBP and EK1 had no obvious negative effects on the original structure and inhibitory mechanism of EK1 but rather improved the intrinsic ability of IBP to bind IgG.

### 3.6. IBP-EK1 Displayed Extended In Vivo Half-Life

**Figure 6:**
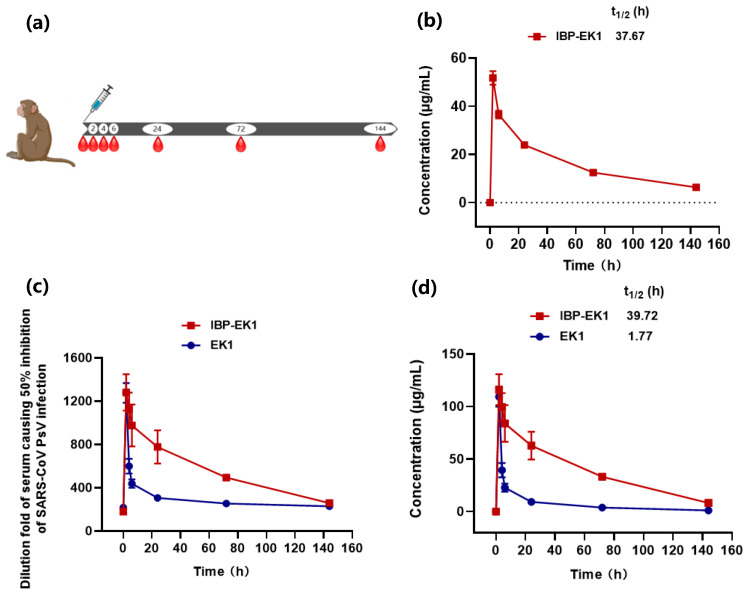
The in vivo half-life of IBP-EK1: (**a**) Rhesus monkeys (*n* = 2) were administered intravenously with a single dose of 10 mg/kg IBP-EK1 (*n* = 2) or EK1 (*n* = 2). Serial blood samples were collected from monkeys that received IBP-EK1 or EK1 before injection and at 2, 4, 6, 24, 72, and 144 h post injection. (**b**) Measured IBP-EK1 concentrations in rhesus monkey sera by the method of HPLC-MS/MS. Sera were diluted 10-fold and then injected into UPLC-Q-TOF-MS for detection. (**c**,**d**) Estimated IBP-EK1 or EK1 concentrations in rhesus monkey sera (**c**), according to dilution folds of serum causing 50% inhibition of SARS-CoV PsV infection (DF-IC_50_) (**d**).

As described above, IBP-EK1 can bind specifically to human and rhesus monkey IgG [56,57]. We thus adopted rhesus monkey as the model to evaluate its in vivo half-life. Rhesus monkeys were administered a single dose of 10 mg/kg IBP-EK1 or EK1 intravenously. Then, blood samples were collected, and sera were separated at different time points (Figure 6a). High-performance liquid chromatography–tandem mass spectrometry (HPLC-MS/MS) has become an important detection technique in the field of drug residue analysis in recent years by integrating efficient separation and multicomponent analysis. Therefore, in order to accurately determine the in vivo half-life of IBP-EK1 or EK1, we quantified their concentrations in sera via HPLC-MS/MS. Serially diluted peptide solutions were first measured to draw their standard curve, respectively, and serum samples were then detected to obtain IBP-EK1 or EK1 concentrations in sera at different time points post injection (Figure 6b and Figure S3b). In addition, as reported through Pearson’s correlation coefficient analysis, a high correlation exists between drug concentration and ex vivo anti-HIV-1 activity of sera from lipopeptide-administered rats [48,49]. To estimate IBP-EK1 or EK1 concentrations in rhesus monkey sera, we first tested the ex vivo inhibitory activities of sera against SARS-CoV PsV infection at different time points, followed by calculating dilution folds causing 50% inhibition of SARS-CoV PsV infection (DF-IC_50_s) (Figure 6c). According to the results, serum samples from rhesus monkeys treated with IBP-EK1 reached inhibition peak at 2 h post injection (about 1300-fold of its IC_50_) and maintained high inhibitory activities for 24 h post injection (over 750-fold of its IC_50_), while those of EK1-treated group decreased quickly (below 450-fold of its IC_50_ after 6 h post injection). Based on their in vitro IC_50_ values and ex vivo anti-SARS-CoV activities, we estimated the concentrations of active inhibitor in sera of rhesus monkey collected at different time points post injection, respectively (Figure 6c). The serum half-lives of IBP-EK1 or EK1 were also calculated using MODFIT software [50] (Figure 6d). The corresponding half-life of IBP-EK1 was 39.7 h, about 21-fold longer than that of EK1 (t_1/2_ = 1.77 h) (Table 3), consistent with the reported value (t_1/2_ = 1.8 h) [32]. These results suggest the half-lives of IBP-EK1 calculated by these two methods were similar and that conjugation of IBP to N-terminus of EK1 could significantly extend the in vivo half-life of EK1.

### 3.7. IBP-EK1 Showed Good In Vivo Safety

**Figure 7:**
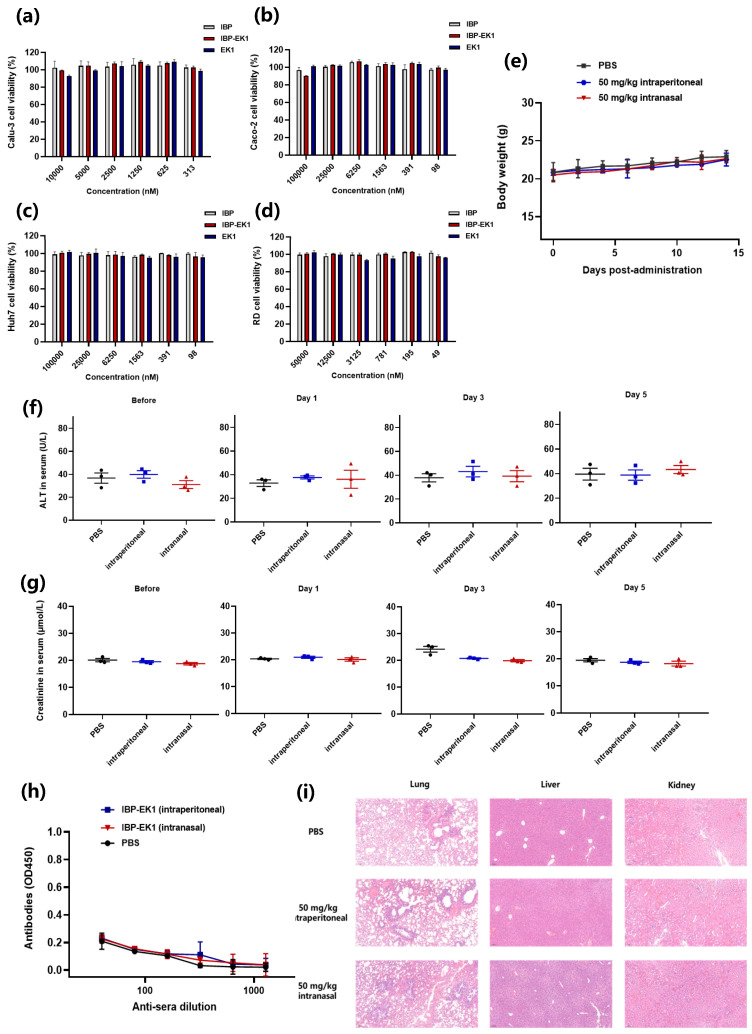
Evaluation of in vitro and in vivo toxicity of IBP-EK1: (**a**–**d**) Cytotoxicity of EK1, IBP, and IBP-EK1 to Calu-3 (**a**), Caco-2 (**b**), Huh-7 (**c**), and RD (**d**) cells. (**e**) Body weight changes in mice treated with 50 mg/kg IBP-EK1 via intraperitoneal injection (i.p.) or intranasal administration. (**f**,**g**) Levels of alanine aminotransferase (ALT) (**f**) and creatinine (**g**) in the sera of mice from each group at indicated time points. (**h**) The titer of specific antibodies (IgG) to IBP-EK1 in the sera of mice 2 weeks post injection or administration. (**i**) H&E staining analysis of lungs, livers, and kidneys from the treated mice 4 weeks post injection or administration. Scale bar, 100 μm.

Safety is an important indicator for drug development and clinical application. To evaluate the in vitro safety of IBP-EK1, we tested the cytotoxicity of peptides on different target cells by CCK8 assay. As shown in Figure 7a–d, IBP-EK1 had no detectable adverse effects on cell viability, even at concentrations up to 100 μM; that is, CC_50_ was more than 100 μM. The corresponding selectivity index (SI = CC_50_/IC_50_) of IBP-EK1 was greater than 100.

We further evaluated the in vivo safety of IBP-EK1. Fifty mg/kg IBP-EK1 was given daily to ICR mice for 5 days by intranasal administration or intraperitoneal injection, and weight changes were recorded for the following 2 weeks. As shown in Figure 7e, mice in the two IBP-EK1-treated groups survived normally with weight changes similar to those of the PBS-treated group. Blood samples were collected on the 1st day before administration and the 1st, 3rd, and 5th days after the last administration, respectively, to measure ALT and creatinine levels in mice sera. At all the indicated time points, ALT (Figure 7f) and creatinine (Figure 7g) levels in the mice sera of the IBP-EK1-treated groups were in the normal range, as well as those in the control (PBS) group. Four weeks after the last administration, mouse organs, including lungs, livers, and kidneys, in each group were collected for histopathological assessment. Compared with the PBS-treated group, IBP-EK1-treated mice showed no cytopathic, necrotic, or inflammatory infiltration, i.e., no pathological abnormalities in their organs (Figure 7i), indicating that IBP-EK1 affords a good in vivo safety profile. Additionally, no IBP-EK1-specific antibodies were detected in mice sera 2 weeks after the last intranasal administration or intraperitoneal injection (Figure 7h), indicating that IBP-EK1 possesses low-immunogenicity advantage, portending its successful clinical application in the future.

### 3.8. IBP-EK1 Synergized with Neutralizing Antibodies Targeting RBD to Robustly Inhibit CoVs In Vitro and Ex Vivo

Since most developed monoclonal neutralizing antibodies belong to IgG isotype, IBP-EK1 can also bind to these neutralizing antibodies. After confirming the extended half-life of IBP-EK1 in rhesus monkeys, we further investigated synergistic antiviral effects of IBP-EK1 in combination with neutralizing antibodies targeting RBD. Based on the binding epitopes, neutralizing antibodies targeting SARS-CoV-2 RBD can be divided into four classes [58,59]. Here, we selected two representative antibodies from different classes to combine with IBP-EK1 (Table S1): REGN10933, a class 1 antibody targeting the receptor-binding motif (RBM) in RBD, as one component of Regeneron’s cocktail therapy [60], and S309, a class 3 antibody targeting RBD (non-RBM), which was developed cooperatively by GSK and Vir technology [61]. S309, identified from memory B cells of individuals infected with SARS-CoV, effectively neutralizes a variety of sarbecoviruses, including SARS-CoV-2 and SARS-CoV, by recognizing highly conserved epitopes among the sarbecovirus subgenus [61] (Table S1).

**Figure 8:**
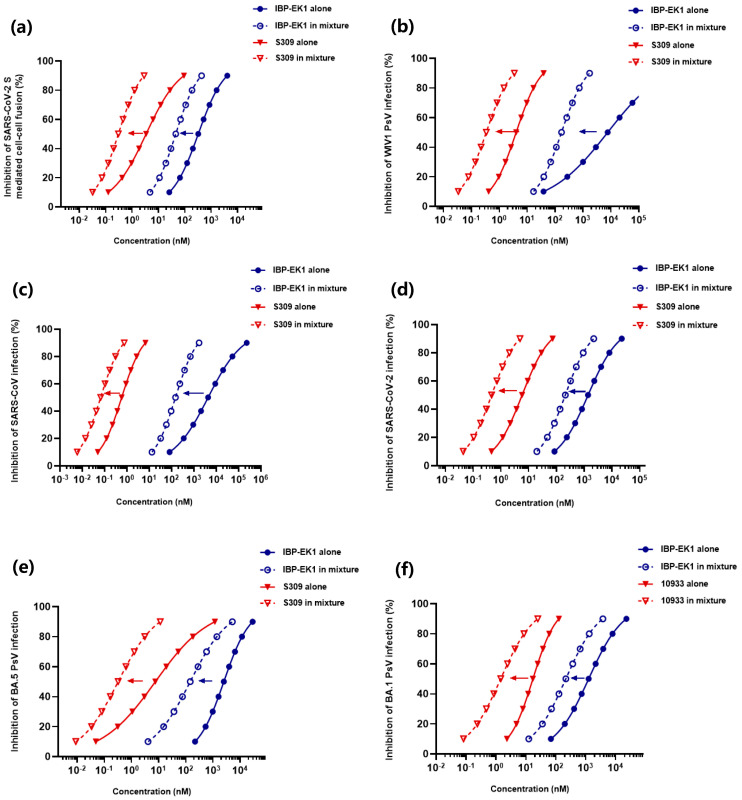
Synergism of IBP-EK1 in combination with neutralizing antibodies to potently combat sarbecoviruses: (**a**) Inhibitory activities of IBP-EK1 alone, S309 alone, or IBP-EK1/S309 in combination against SARS-CoV-2 S-mediated cell–cell fusion. (**b**–**e**) Inhibitory activities of IBP-EK1 alone, S309 alone, or IBP-EK1/S309 in combination against WIV1-CoV (**b**), SARS-CoV (**c**), SARS-CoV-2 prototype (**d**), and BA.5 (**e**) PsV infection. (**f**) Inhibitory activities of IBP-EK1 alone, 10,933 alone, or IBP-EK1/10933 in combination against BA.1 PsV infection. Each sample was tested in triplicate, and each experiment was repeated twice.

As reported before and described above, neutralizing antibodies targeting the S1 subunit may block viral membrane fusion by inhibiting S protein rearrangement [62], while IBP-EK1 blocks viral membrane fusion by interacting with the HR1 region on the S2 subunit. To investigate the synergistic effects of IBP-EK1 and neutralizing antibody, as represented by S309, we detected the inhibitory activities of S309 alone, IBP-EK1 alone, and S309/IBP-EK1 in combination against SARS-CoV-2 S-mediated cell–cell fusion, respectively, and found potent synergistic effect between S309 and IBP-EK1 with the combination index (CI) causing 50% fusion-inhibition of 0.23, and dose reductions in S309 and IBP-EK1 were 11.3- and 7.1-fold, respectively (Figure 8a and Table S2).

Consistently, on pseudotyped infection models of SARS-CoV, WIV1-CoV, and SARS-CoV-2 and its variants, IBP-EK1 in combination with S309 showed strong synergistic effects with CI values inhibiting 50% PsV infection, ranging from 0.10 to 0.24, which significantly improved the IC_50_ value of S309, ranging from 8- to 24-fold (Figure 8 and Table 4). In order to investigate whether IBP-EK1 has universal synergistic effects with antibodies targeting RBD, we then evaluated the synergistic effect of IBP-EK1 and 10,933 against SARS-CoV-2 and its variants. As expected, IBP-EK1/10933 also showed significant synergism (Figure 8 and Table 4), suggesting that IBP-EK1 could broadly synergize with neutralizing antibodies targeting diverse epitopes of RBD.

**Figure 9:**
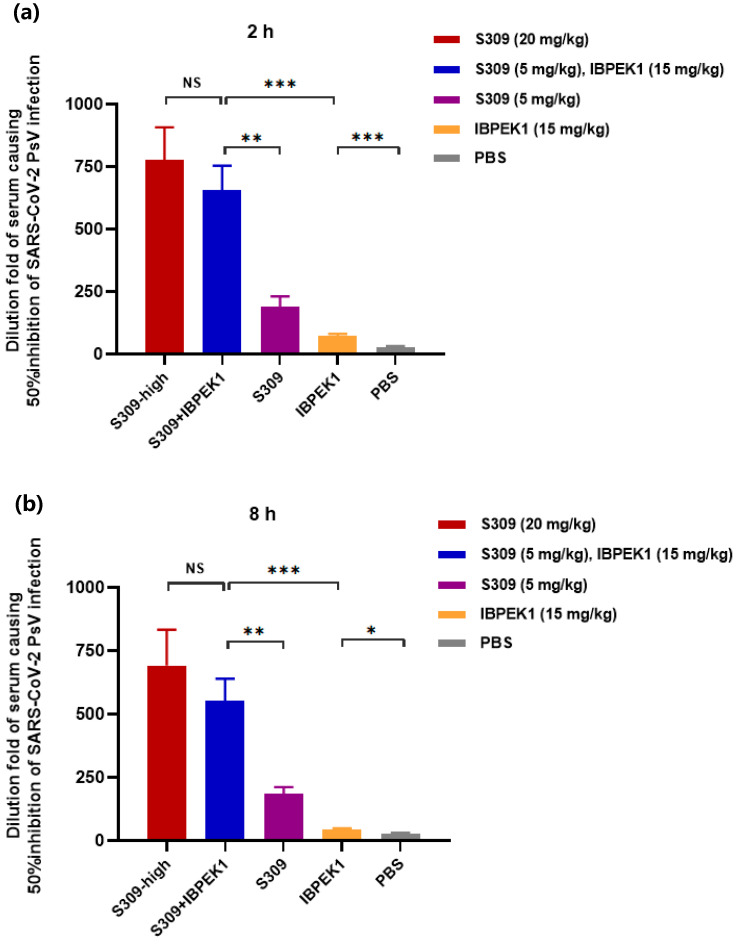
The ex vivo synergistic effect of IBP-EK1 in combination with S309 against SARS-CoV-2 infection: (**a**,**b**) Sera were collected from mice 2 h (**a**) and 8 h (**b**) post injection via i.p. with S309 (20 mg/kg), S309 (5 mg/kg)/IBP-EK1 (15 mg/kg) in combination, IBP-EK1 (15 mg/kg), or S309 (5 mg/kg), respectively. A representative example of two independent experiments is shown. The dilution folds of serum causing 50% inhibition of SARS-CoV-2 infection (DF-IC_50_s) were calculated. Each sample was tested in triplicate, and data are presented as mean ± SD. Unpaired, 2-tailed *t*-test was performed. NS, *p* > 0.05; * *p* < 0.05, ** *p* < 0.01, and *** *p* < 0.001.

To explore the possible in vivo synergistic effect of IBP-EK1 in combination with neutralizing antibodies, we evaluated the ex vivo anti-SARS-CoV-2 activities of mouse sera administered with IBP-EK1 in combination with S309. According to their in vitro IC_50_ values against SARS-CoV-2 PsV, as described above, the appropriate molar concentration ratio between IBP-EK1 and S309 is about 3:1. Mice were administered intraperitoneally with 20 mg/kg or 5 mg/kg S309 alone, 15 mg/kg IBP-EK1 alone, and S309 (5 mg/kg)/IBP-EK1 (15 mg/kg), respectively, and mouse sera were collected 2 h and 8 h later. Then, inhibitory activities against SARS-CoV-2 PsV of mouse sera from different groups were tested, and dilution folds causing 50% inhibition of SARS-CoV-2 PsV infection (DF-IC_50_s) were calculated. As demonstrated in Figure 9a, 2 h after administration, sera from the combined S309/IBP-EK1 group (DF-IC_50_ = 656) showed significantly higher inhibition when compared with sera from the S309 (5 mg/kg) group (DF-IC_50_ = 189) and IBP-EK1 (15 mg/kg) group (DF-IC_50_ = 73), as well as sera from the high-dose S309 (20 mg/kg) group (DF-IC_50_ = 777). Similarly, 8 h after administration, no significant difference was observed between the inhibitory activities of sera from the S309 (20 mg/kg) group (DF-IC_50_ = 690) and the combined S309/IBP-EK1 group (DF-IC_50_ = 550), which still showed significantly stronger inhibition compared with sera from the S309 (5 mg/kg) group (DF-IC_50_ = 185) and IBP-EK1 (15 mg/kg) group (DF-IC_50_ = 44) (Figure 9b). These results show that IBP-EK1 combined with S309 had an effective ex vivo synergistic antiviral effect, which compensated for the decreased activities caused by lower concentrations of S309 or IBP-EK1. The results also imply that the combination of IBP-EK1 with neutralizing antibodies can be expected to synergize during in vivo circulation.

## 4. Discussion

The application of anti-HCoV fusion inhibitory peptides is limited by their rapid clearance and short half-life in vivo. Although various long-acting strategies have been widely used, most strategies may interfere with the original functions of peptides [39]. To solve this problem, we selected IBP (Fc-III) with low molecular weight, simple structure, and nonbacterial origin to modify fusion inhibitory peptides. In fact, IgG-binding peptides (IgGBPs) are a class of peptides targeting IgG (primarily its Fc domain) [63]. In addition to the IBP (Fc-III) adopted in this study, a number of studies have also screened out short peptides targeting different sites of the IgG Fc domain. These short peptides are characterized by low toxicity, high stability, low cost, and selectable affinity [41], such as linear hexapeptides targeting the Fc C_H_3 region (HWRGWV [64], HYFKFD [65], and HFRRHL [66]) or octopeptides targeting the Fc C_H_2/C_H_3 interface (FYWHCLDE [67], FYCHWALE [68]). Fc-III-derived cyclic peptidomimetic FcBP-2 [69] and bicyclic derivative Fc-III-4C [70] were subsequently reported with improved binding affinity for the IgG Fc domain, thus enabling the development of a variety of long-acting strategies for peptide drugs. Nevertheless, further optimization of IgGBP affinity for Fc C_H_2/C_H_3 interface, or generation and utilization of ligands with high affinity to other binding sites on IgG Fc, deserves in-depth investigation.

In this study, we developed an IBP-conjugated fusion inhibitor, IBP-EK1, which effectively inhibited in vitro PsV infection of SARS-CoV-2 variants and showed dose-dependent inhibition of authentic Delta variant proliferation. IBP-EK1 also showed broadly inhibitory activities against cell–cell fusion and PsV infection mediated by the S protein of divergent HCoVs and replication of live HCoVs. Furthermore, IBP-EK1 exhibited in vivo prophylactic and therapeutic effects against HCoV-OC43 infection in newborn mice. Subsequently, our results indicate that IBP conjugation did not affect the structure or inhibitory mechanism of EK1. Of note, IBP conjugation with EK1 significantly improved the binding and affinity of IBP to human or monkey IgG and substantially prolonged the serum half-life of EK1 in vivo. In addition, IBP-EK1 combination with monoclonal neutralizing antibodies targeting SARS-CoV-2 RBD exhibited robust synergistic antiviral effects, both in vitro and ex vivo, significantly reducing doses of IBP-EK1 and antibodies.

The application of peptide fusion inhibitors is often limited by their physicochemical properties, such as molecular weight, charge/hydrophobicity, and in vivo stability [33]. HCoVs are mostly transmitted through respiratory infections, and viral load in the respiratory tract was found to be associated with the disease severity of COVID-19 patients [71,72]. Peptide inhibitors administered intranasally can block the HCoV entry process as the first step of viral invasion, making these inhibitors promising for clinical application. Although intranasal administration can avoid renal filtration and hepatic first-pass effect, diverse enzymes in the nasal mucosa, such as aminopeptidases and proteases, impact the in vivo stability of peptide drugs [73]. Studies have shown that cyclic peptides have relatively restricted conformations with reduced protease recognition and higher stability in vivo [74]. Since IBP itself appears cyclic, we studied the effect of IBP conjugation on in vivo stability of EK1 and tested the tolerance of EK1 to protease hydrolysis. Results demonstrate that samples of IBP-EK1 treated with trypsin (Figure S5a) or proteinase K (Figure S5b) had significantly better inhibitory activities than those of EK1 against SARS-CoV-2 PsV infection at different incubation time points, indicating that IBP conjugation could improve the original tolerance of EK1 to protease hydrolysis. This result suggests that IBP-EK1 may display better in vivo stability in environments with proteases.

As described above, IBP conformation is somewhat restrictive [74], and conjugation with EK1 increases its original hydrophilicity and stability (Table S3). Hence, IBP-EK1 may fit the C_H_2/C_H_3 interface of the IgG Fc domain more appropriately. This explains why IBP-EK1 dissociation from IgG is more problematic when compared with IBP, since the Koff value of IBP-EK1 is lower and its affinity is higher when compared with IBP alone (Table S4). For further investigation and confirmation, the crystal structure of IBP-EK1 in complex with IgG will be analyzed in future studies.

Since SARS-CoV-2 presents as a systemic, multitissue, and organ-specific infection in patients [75], neutralizing antibodies administered by intravenous infusion can effectively reduce the in vivo viral titer and avoid excessive immune response, such as cytokine storm, during the period of transition between quiescent and proliferating states of adaptive immune response [76,77]. Besides, for some severe patients unable to generate an effective adaptive immune response, neutralizing antibody therapies are also expected to play a therapeutic role [78], especially with extended half-life through Fc-silencing technologies [79]. However, the high cost of such therapies limits their use, especially against Omicron subvariants able to effectively evade immunity.

Compared with the expression and purification of monoclonal antibodies, the production of peptides is simpler and faster, with a lower cost. As demonstrated above, IBP-EK1, which is derived from IBP (Fc-III) conjugation, increases its apparent molecular weight by binding to IgG in serum, thereby extending its half-life in vivo. Therefore, IBP-EK1, a long-acting, broad-spectrum HCoV fusion inhibitory peptide, can be administered via intravenous infusion, similar to that of therapeutic neutralizing antibodies. It has been reported that most neutralizing monoclonal antibodies need to be administered in combination to reduce doses and limit the emergence of drug-resistant variants, and their isotype is mainly IgG. Therefore, the combination of IBP-EK1 with neutralizing monoclonal antibodies, such as S309, targeting conserved sites on RBD is expected to circulate in vivo and interact with each other to generate a synergistic effect over a prolonged half-life [80], thus reducing doses of antibodies and peptides, as well as therapeutic costs.

Based on the K_D_ values obtained and concentrations adopted in this study, we can theoretically deduce the feasible synergism between IBP-EK1 and S309, whereby their combined effect is greater than the sum of their separate effects in vivo, as follows. According to the results of the BLI assay, the binding affinity of IBP-EK1 to human IgG is about 200 nM, which is sufficient for their in vivo interaction. We liken this interaction to physiological receptor–ligand affinity, such as, for example, that between PD-1 and PD-L1 with 112~526 nM affinity [81]. Furthermore, the concentration of IBP-EK1 in rhesus monkey sera 24 h after 10 mg/kg administration was about 24 μg/mL, i.e., 4 μM, which theoretically meets the dose required for combination therapy. Supposing S309 is dosed at 5 mg/kg, an adult needs about 300 to 400 mg of the antibody, consistent with the dosages of currently used neutralizing antibody drugs; and in the case of slow attenuation, its serum concentration may be about 0.4 μM (with human blood volume estimated about 4 L) 24 h after 5 mg/kg administration. Therefore, if S309 (5 mg/kg) and IBP-EK1 (15 mg/kg) are dosed in combination, they may act synergistically to block the CoV entry process during a certain period of time after administration.

Recently, broadly neutralizing antibodies (bNAbs) targeting conserved sites on HCoV S2 subunit have been identified, such as 76E1 targeting fusion peptide (FP) and S2P6 targeting stem helix (SH) [82,83]. The synergistic effect of combining IBP-EK1 with these bNAbs targeting the HCoV S2 subunit could be further explored to provide a reserve for newly emerging or re-emerging CoV outbreaks in the future.

## 5. Conclusions

In conclusion, our study focused on the long-acting strategy of IBP conjugation to extend in vivo half-lives of CoV peptide fusion inhibitors, as represented by EK1. Our results demonstrate that the strategy of IBP conjugation at N-terminus of EK1 could maintain, or even improve, the anti-HCoV activities of EK1, significantly extending its original in vivo half-life and generating effective synergism with neutralizing antibodies. The results also suggest that IBP-EK1 could be administered via the intranasal route or intravenous infusion (in combination with neutralizing antibodies) for development into a long-acting, broad-spectrum CoV peptide fusion inhibitor. In addition to EK1 mentioned here, this long-acting strategy based on IBP conjugation could also be generalized to fusion (entry) inhibitory peptides against other type I enveloped viruses, such as HIV-1 [47].

## Figures and Tables

**Table 1:** IC_50_ Values of IBP-EK1 and EK1 against SARS-CoV-2 Variants PsV Infection.

SARS-CoV-2 Variants	IC_50_ (nM) against Pseudotyped SARS-CoV-2 Variants on Caco-2 Cells			IC_50_ (nM) against Pseudotyped SARS-CoV-2 Variants on Calu-3 Cells		
	IBP	IBP-EK1	EK1	IBP	IBP-EK1	EK1
N501Y	>50,000	718	737	>50,000	266	347
B.1.1.7	>50,000	457	477	>50,000	241	259
B.1.351	>50,000	677	719	>50,000	281	349
P.1	>50,000	338	428	>50,000	171	246
B.1.617.2	>50,000	389	527	>50,000	296	303
C.37	>50,000	722	791	>50,000	289	357
BA.1	>50,000	301	334	NA		
BA.2	>50,000	399	544			
BA.2.12.1	>50,000	527	592			
BA.2.75	>50,000	221	248			
BA.5	>50,000	564	690			
BF.7	>50,000	257	260			
BA.4.6	>50,000	195	201			
XBB	>50,000	330	366			
BQ.1.1	>50,000	342	378			

NA, not applicable. Each experiment was repeated three times. Colors from red to green correspond to IC_50_ values from high to low.

**Table 2:** IC_50_ Values of IBP-EK1 and EK1 against the Tested CoVs (Mainly HCoVs).

Coronaviruses	Genus	Receptor (Reported)	IC_50_ (nM) against Pseudotyped CoVs on Caco-2 Cells			IC_50_ (nM) against Pseudotyped CoVs on Calu-3 Cells			IC_50_ (nM) against Live CoV Infection			IC_50_ (nM) against Cell-Cell Fusion Mediated by S Proteins of CoVs		
			IBP	IBP-EK1	EK1	IBP	IBP-EK1	EK1	IBP	IBP-EK1	EK1	IBP	IBP-EK1	EK1
SARS-CoV	β	ACE2	>50,000	1301	1441	>50,000	18	24	ND			>50,000	229	426
MERS-CoV	β	DPP4	>50,000	522	885	>50,000	76	77	ND			>50,000	376	492
SARS-CoV-2	β	ACE2	>50,000	512	590	>50,000	180	253	>50,000	204	296	>50,000	512	590
WIV1-CoV	β	ACE2	>50,000	6047	6985	NA			ND			>50,000	306	588
HCoV-OC43	β	9-O-Ac-Sias	>50,000	3574	3611	NA			>50,000	744	764	>50,000	829	1161
HCoV-NL63	α	ACE2	>50,000	5641	5934	>50,000	440	537	ND			>50,000	212	721
HCoV-229E	α	hAPN	>50,000	3344	3475	NA			>50,000	4139	7592	>50,000	478	623

NA, not applicable; ND, not detected. Each experiment was repeated three times. Colors from red to green correspond to IC_50_ values from high to low.

**Table 3:** In Vivo Pharmacokinetic Parameters of IBP-EK1 Measured with Two Methods, Respectively, and Calculated by MODFIT Software.

Parameter	IBP-EK1 (UPLC-Q-TOF-MS)	IBP-EK1 (Estimated)
t_1/2_ (h)	37.67	39.72
T_max_ (h)	2	2
C_max_ (μg/mL)	51.73	116.09
AUC (μg/mL × h)	2207.03	5228.98

**Table 4:** Combination Index (CI) and Dose Reduction Values of Inhibiting 50% PsV Infection by IBP-EK1 Combination with Neutralizing Antibodies.

IBP-EK1 Combination with Antibody against Sarbecoviruses	CI	Antibody			IBP-EK1		
		IC_50_ (nM)		Dose Reduction (n-Fold)	IC_50_ (nM)		Dose Reduction (n-Fold)
		Alone	In Mixture		Alone	In Mixture	
**10,933**							
B.1.351 (Beta)	0.32	0.51	0.11	4.5	1673	168.8	9.9
P.1 (Gamma)	0.38	0.46	0.13	3.6	1556	154.9	10.1
BA.1	0.26	17.22	1.44	11.9	1256	216.6	5.8
**S309**							
SARS-CoV-2	0.23	5.81	0.46	12.6	1381	208.4	6.6
B.1.117 (Alpha)	0.22	3.10	0.24	12.8	801.0	109.2	7.3
B.1.351 (Beta)	0.23	1.20	0.15	7.9	670.6	68.3	9.8
P.1 (Gamma)	0.24	4.01	0.26	15.6	647.6	115.7	5.6
B.1.617.2 (Delta)	0.14	1.20	0.12	10.2	1533	70.5	21.7
BA.1	0.19	3.28	0.30	11.0	1416	134.7	10.5
BA.5	0.10	7.62	0.32	23.5	2520	145.8	17.3
XBB	0.14	5.28	0.43	12.2	1217	70.8	17.2
BQ.1.1	0.20	52.98	3.11	17.0	1123	155.7	7.2
SARS-CoV	0.15	0.59	0.07	8.8	4358	150.3	29.0
WIV1-CoV	0.11	4.03	0.34	11.8	7713	170.5	45.3

Each experiment was repeated twice. Colors from red to green in each line correspond to CI values from high to low and dose reduction values from low to high, respectively.

## Data Availability

The raw data of this paper are available on request from the corresponding author.

## Supplementary Materials

The following supporting information can be downloaded at: https://www.mdpi.com/article/10.3390/biom13091283/s1: Figure S1. Inhibitory activities of IBP-EK1 against SARS-CoV-2 variants PsV infection on Caco-2 cells; Figure S2. Pathology sections of mouse brain tissues of each group 5 days after administration; Figure S3. Homology between IgG1 Fc region of homo sapiens and rhesus monkey, and measured EK1 concentrations in sera by HPLC-MS/MS; Figure S4. Binding of IBP, EK1 and IBP-EK1 to rhesus monkey IgG; Figure S5. Tolerance of IBP-EK1 to protease digestion; Table S1. IC_50_ values of S309 and 10933 against PsV infection of sarbecoviruses; Table S2. Combination index and dose reduction values of inhibiting SARS-CoV-2 S-mediated cell-cell fusion by IBP-EK1 combination with S309; Table S3. Prediction of the physicochemical properties of IBP-EK1 and IBP; Table S4. Rate constants and K_D_ values of IBP-EK1 and IBP to human or rhesus monkey IgG.
