# Supplementary material for: IgG Fc-Binding Peptide-Conjugated Pan-CoV Fusion Inhibitor Exhibits Extended In Vivo Half-Life and Synergistic Antiviral Effect When Combined with Neutralizing Antibodies
Source: Biomolecules. 2023 Aug 22;13(9):1283. doi: 10.3390/biom13091283 (PMC10526447; doi:10.3390/biom13091283)
Supplement: Supplementary file 1 [file biomolecules-13-01283-s001.zip › biomolecules-2466445-supplementary.pdf]

biomolecules

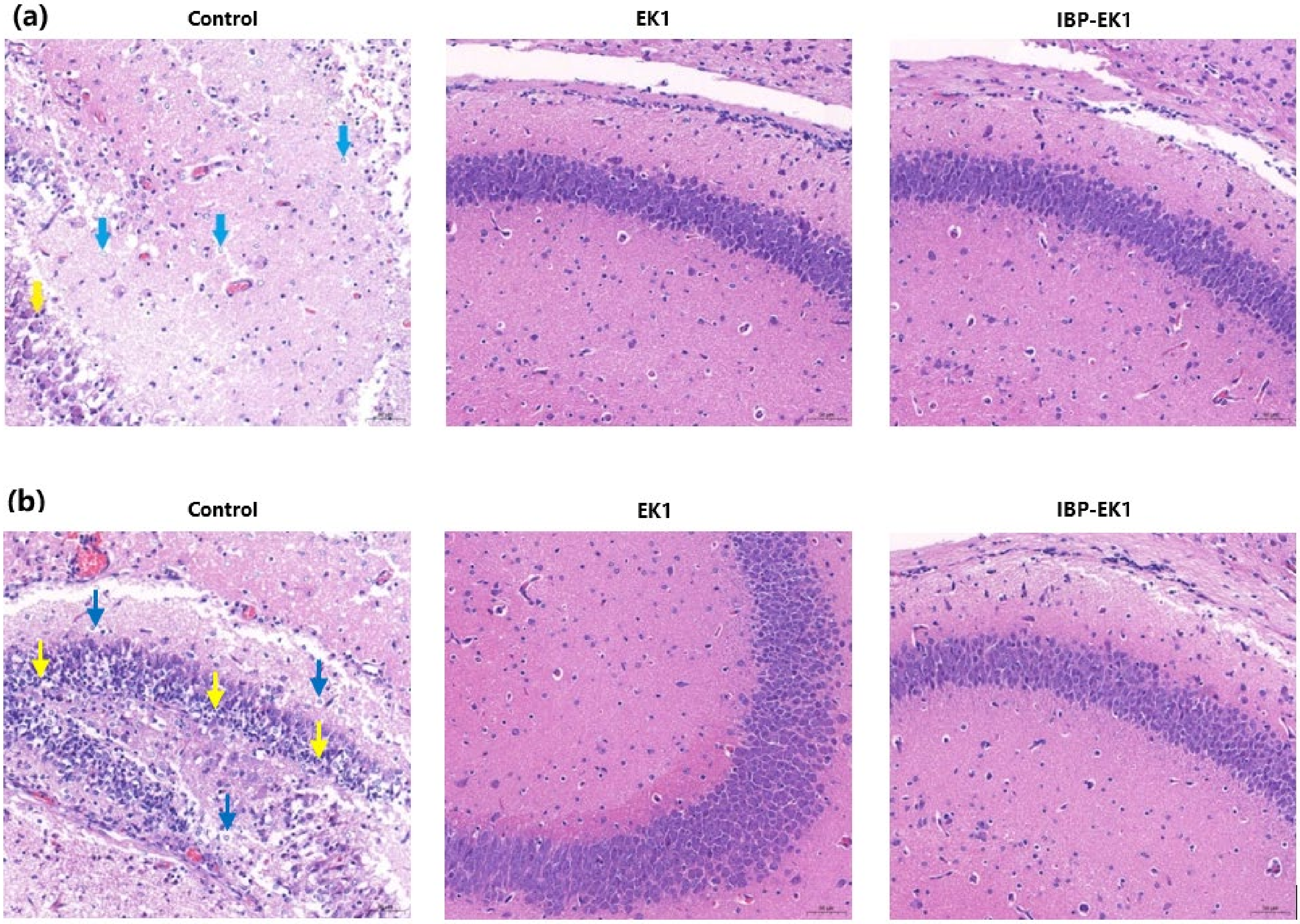

Figure S2. Pathology sections of mouse brain tissues of each group 5 days after administration. (**a**) Pathology sections of mouse brain tissues of each group 5 days after prophylactic administration. (**b**) Pathology sections of mouse brain tissues of each group 5 days after therapeutic administration. Scale bar, 50 μm.

(a)

```
Identities            Positives             Gaps
200/223(90%)          210/223(94%)          0/223(0%)

5W5L 225  TCPPCPAPELLGGPSVFLFPPKPKDTLMISRTPEVTCVVVDVSQEDPDVKFN
6D4E 225  .........AA.AS.............................A...E....

5W5L      WYVNGAEVHHAQTKPRETQYNSTYRVVSVLTVTHQDWLNGKEYTCKVSNKAL
6D4E      ...D.V...N.K.....E..............L..........K........

5W5L      PAPIQKTISKDKGQPREPQVYTLPPSREELTKNQVSLTCLVKGFYPSDIVVE
6D4E      .SS.E.....A................D.....................A..

5W5L      WESSGQPENTYKTTPPVLDSDGSYFLYSKLTVDKSRWQQGNVFSCSVMHEAL
6D4E      ...N.....N.............F............................

5W5L      HNHYTQKSLSVSPGK 447
6D4E      ..........L.... 447
```

(b)

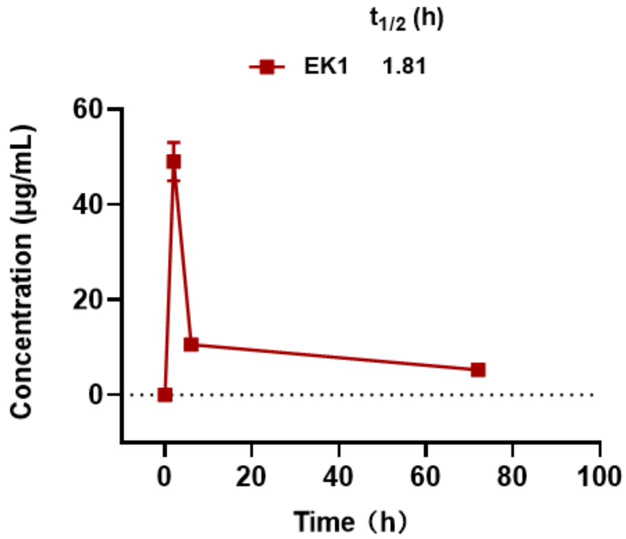

Figure S3. Homology between IgG1 Fc region of homo sapiens and rhesus monkey, and measured EK1 concentrations in sera by HPLC-MS/MS. **(a)** Homology between IgG1 Fc region of homo sapiens and rhesus monkey. Fasta files of IgG1 Fc CH2-CH3 region of human (PDB ID: 5W5L) and rhesus monkey (PDB ID: 6D4E) were exported from PDB, respectively, and aligned by blastp tool. Residues in red boxes are critical residues binding to IBP. **(b)** Measured EK1 concentrations in rhesus monkey sera by the method of HPLC-MS/MS. Sera were diluted 10-fold and then injected into UPLC-Q-TOF-MS for detection.

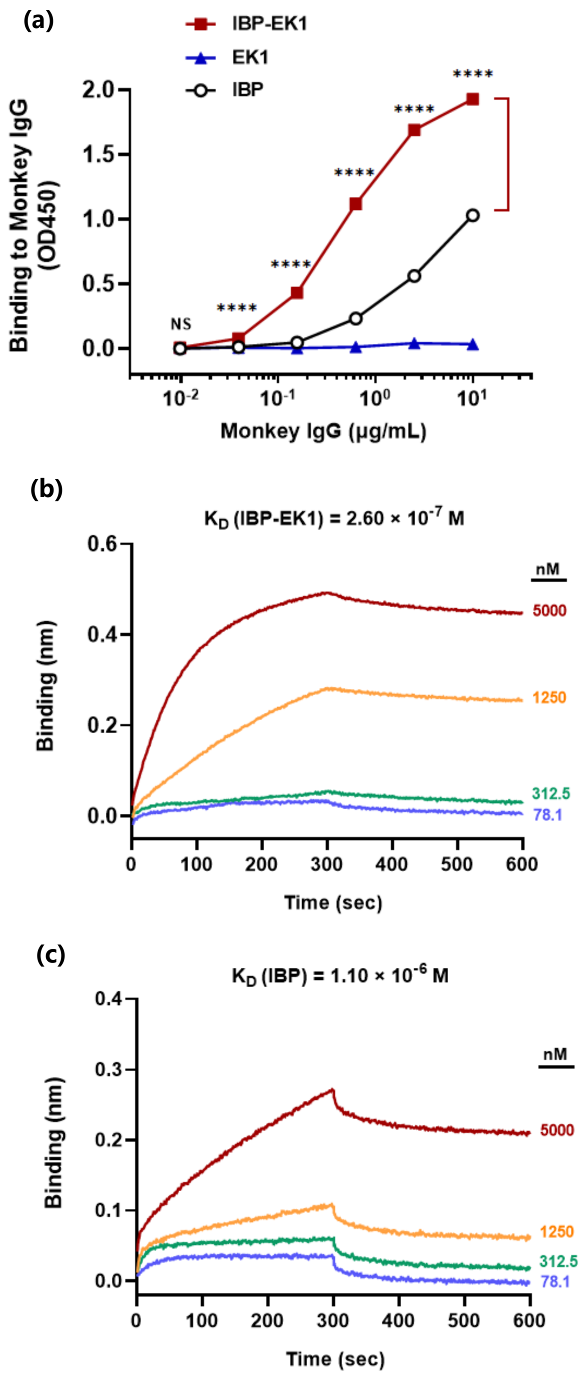

Figure S4. Binding of IBP, EK1 and IBP-EK1 to rhesus monkey IgG, as etected by ELISA (**a**) and BLI (**b**d -**c**). NS, $p > 0.05$; **** $p < 0.0001$. In the BLI assay, 10 μg/mL IgG were immobilized at the surface of Fc biosensors, and then diluted peptide solutions were loaded. Data were analyzed and globally fit with the ForteBio software. Equilibrium dissociation constants (K_D) are reported above the plot.

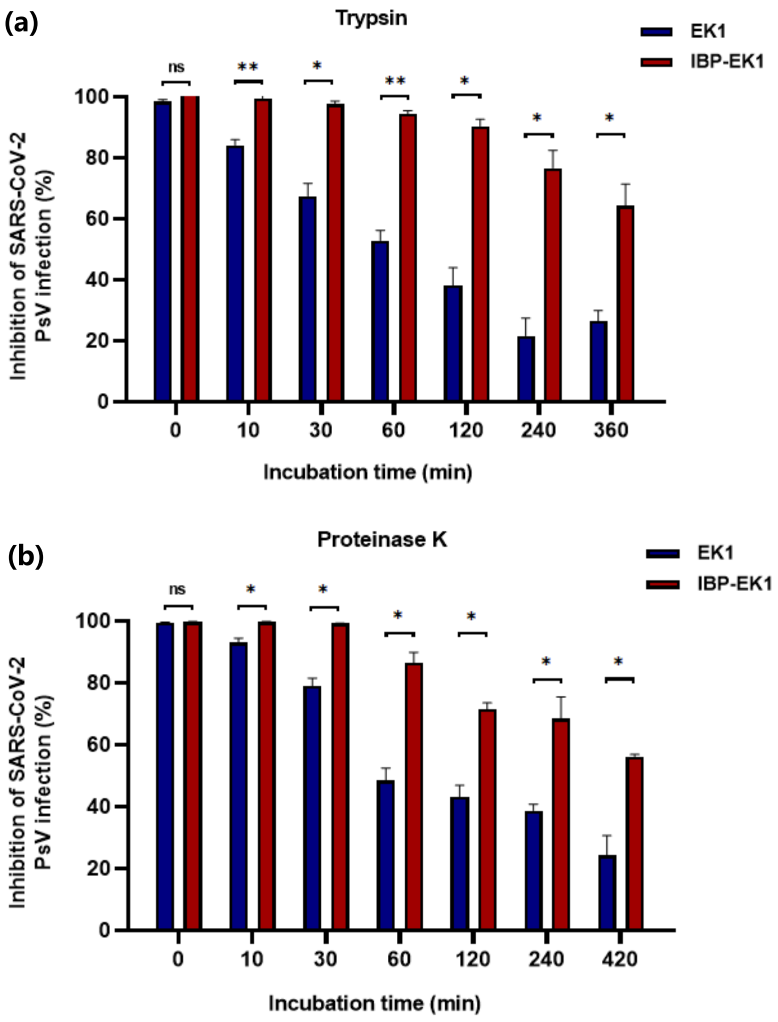

Figure S5. Tolerance of IBP-EK1 to protease digestion. (**a**) Inhibitory activities against SARS-CoV-2 PsV infection of EK1 and IBP-EK1 samples incubated with 25 µg/mL trypsin for different time. (**b**) Inhibitory activities against SARS-CoV-2 PsV infection of EK1 and IBP-EK1 samples incubated with 100 µg/mL proteinase K for different time. Unpaired, 2-tailed -*t* test was performed. No significance (ns), $p > 0.05$; * $p < 0.05$, ** $p < 0.01$. A representative example of two independent experiments was shown.

Table S1. IC_{50} values of S309 and 10933 against PsV infection of sarbecoviruses (mainly SARS-CoV-2 variants). ND, not detected. Each experiment was repeated twice.

Sarbecoviruses	IC_{50} (ng/mL) against pseudotyped sarbecoviruses on Caco-2 cells	
	S309	10933
SARS-CoV	86	ND
WIV1-CoV	769	ND
SARS-CoV-2 prototype	722	27
B.1.1.7	134	12
B.1.351	90	114
P.1	150	137

B.1.617.2	284	20
BA.1	323	1859
BA.2	482	>10,000
BA.2.12.1	574	>10,000
BA.2.75	640	>10,000
BA.5	706	>100,000
BA.4.6	1340	>100,000
BF.7	1098	>100,000
XBB	1278	>100,000
BQ.1.1	4180	>100,000

Table S2. Combination index and dose reduction values of inhibiting SARS-CoV-2 S-mediated cell-cell fusion by IBP-EK1 combination with S309.

% Inhibition for IBP-EK1 in combination with S309 against cell-cell fusion	CI	S309			IBP-EK1		
		Concentration (nM)		Dose reduction	Concentration (nM)		Dose reduction
		Alone	Mix		Alone	Mix	
**10**	**0.44**	0.13	0.03	**3.9**	25.7	4.8	**5.4**
**20**	**0.34**	0.42	0.07	**5.8**	65.6	11.0	**6.0**
**30**	**0.29**	0.96	0.13	**7.5**	122.2	19.1	**6.4**
**40**	**0.26**	1.86	0.20	**9.3**	203.6	30.0	**6.8**
**50**	**0.23**	3.42	0.30	**11.3**	325.0	45.5	**7.1**
**60**	**0.21**	6.30	0.46	**13.7**	519.0	68.9	**7.5**
**70**	**0.18**	12.25	0.72	**17.0**	864.3	108.4	**8.0**
**80**	**0.16**	27.59	1.25	**22.0**	1610	188.2	**8.6**
**90**	**0.14**	93.55	2.88	**32.5**	4105	432.1	**9.5**

Table S3. Prediction of the physicochemical properties of IBP-EK1 and IBP. GRAVY, Grand average of hydropathicity; if the value is negative, the protein/peptide is hydrophilic, otherwise hydrophobic. The ProtParam tool (https://web.expasy.org/ protparam/) was adopted to predict these properties.

Properties	IBP-EK1	IBP	EK1
Instability index	29.30 (stable)	46.72 (unstable)	22.73 (stable)
GRAVY	-0.206	0.423	-0.433
Aliphatic index	111.43	90.00	119.17

Table S4. Rate constants and K_D values of IBP-EK1 and IBP to human or rhesus monkey IgG.

**Constants**	**human IgG**		**rhesus monkey IgG**	
	IBP-EK1	IBP	IBP-EK1	IBP
Kon (Ms^{-1})	3.30×10^{3}	2.80×10^{3}	2.04×10^{3}	1.27×10^{3}
Koff (s^{-1})	7.17×10^{-4}	2.87×10^{-3}	5.29×10^{-4}	1.40×10^{-3}
K_D (M)	2.17×10^{-7}	1.03×10^{-6}	2.60×10^{-7}	1.10×10^{-6}
